# Coordination polymers of 5-substituted isophthalic acid†
†Electronic supplementary information (ESI) available. CCDC 1417516–1417520 contain the supplementary crystallographic data for this paper. For ESI and crystallographic data in CIF or other electronic format see DOI: 10.1039/c5ce02091c



**DOI:** 10.1039/c5ce02091c

**Published:** 2015-12-21

**Authors:** Laura J. McCormick, Samuel A. Morris, Alexandra M. Z. Slawin, Simon J. Teat, Russell E. Morris

**Affiliations:** a EaSTCHEM School of Chemistry , University of St Andrews , North Haugh , St Andrews , Fife , Scotland KY16 9ST , UK . Email: ljm22@st-andrews.ac.uk ; Fax: +44 (0)1334 463808 ; Tel: +44 (0)1334 463776; b Advanced Light Source , Berkeley Laboratory , 1 Cyclotron Road , Berkeley , California 94720 , USA

## Abstract

The synthesis and characterisation of five coordination polymers – Ni_2_(mip)_2_(H_2_O)_8_·2H_2_O (**1**), Zn_6_(mip)_5_(OH)_2_(H_2_O)_4_·7.4H_2_O (**2**), Zn_6_(mip)_5_(OH)_2_(H_2_O)_2_·4H_2_O (**3**), Mn(HMeOip)_2_ (**4**), and Mn_3_(tbip)_2_(Htbip)_2_(EtOH)_2_ (**5**) – are reported. Preliminary nitric oxide release data on compounds **2** and **3** are also given.

## Abstract 2

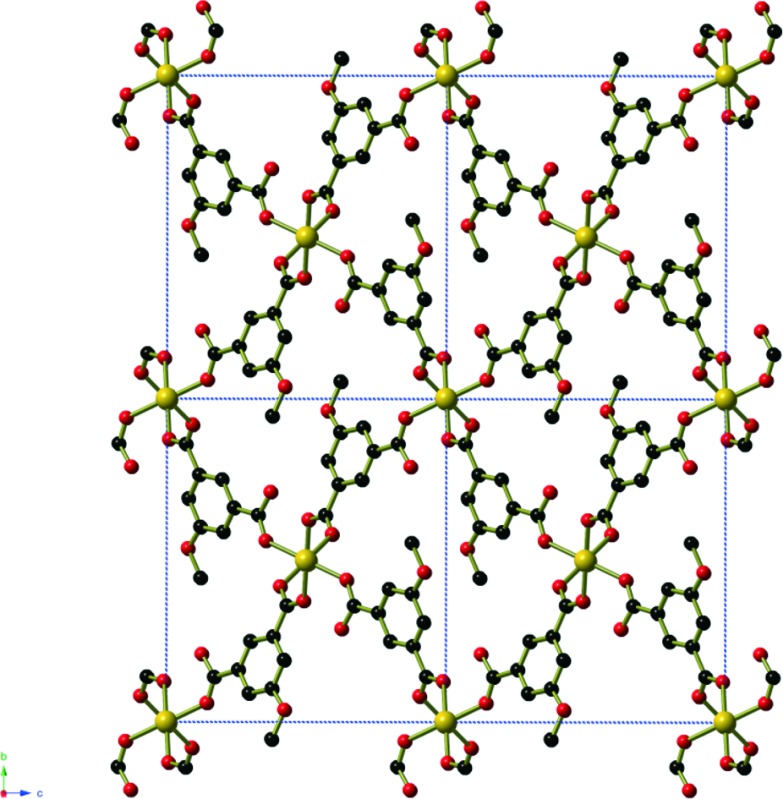
Crystallographic investigations were conducted into coordination polymers derived from 5-methyl, 5-methoxy and 5-*tert*-butyl isophthalic acids. Preliminary nitric oxide storage and release properties are also reported.

## Introduction

Design and synthesis of new coordination polymers is currently a burgeoning field, due to the ability to tailor the properties of these materials by adjusting their structure.^1^ One such property that is presently garnering interest is the use of porous coordination polymers (also known as metal–organic frameworks or MOFs) as gas storage and delivery materials, exploiting the large surface areas of these materials coupled, in many cases, with binding gas molecules to coordination sites from which a coordinated solvent molecule has been removed. The use of these materials as gas delivery systems is advantageous for both energy storage applications^2^ (adsorbed gas molecules do not contribute to storage vessel pressure, allowing large volumes of gas to be stored without the complications of a high pressure container) and medical applications^3^ (coordinated gas molecules may be displaced by water, allowing for targeted gas delivery without the systemic exposure necessitated by use of delivery of these gases *via* inhalation or ingestion^4^). A medicinal gas of particular interest is nitric oxide (NO), which has been shown to play a role in a variety of biological processes including wound healing and thrombosis prevention, as well as being an anti-bacterial agent.^5^

Coordination polymers based on 5-substituted isophthalate ligands have been shown to exhibit catalytic,^6^ magnetic,^7^ gas storage and flexible framework^8^ properties. Despite being very closely related to the ligands used in some of the best known and widely studied MOFs such as terephthalic acid (MOF-5,^9^ UiO-66,^10^ MIL-101^11^) and trimesic acid (HKUST-1,^12^ MIL-100^13^), surprisingly few coordination polymers derived from the commercially available 5-methyl^14^ and *tert*-butyl^15^ isophthalic acid or the readily synthesised 5-alkoxy^16^ or aminoalkyl^17^ isophthalate ligands have been reported that do not contain additional pyridine- or imidazole-based bridging co-ligands. Herein we reported the synthesis and characterisation of 5 novel coordination polymers derived from the 5-methyl (H_2_mip, I), methoxy (H_2_MeOip, II) and *tert*-butyl (H_2_tbip, III) isophthalic acids, namely Ni_2_(mip)_2_(H_2_O)_8_·2H_2_O (**1**), Zn_6_(mip)_5_(OH)_2_(H_2_O)_4_·7.4H_2_O (**2**), Zn_6_(mip)_5_(OH)_2_(H_2_O)_2_·4H_2_O (**3**), Mn(HMeOip)_2_ (**4**), and Mn_3_(tbip)_2_(Htbip)_2_(EtOH)_2_ (**5**). Preliminary investigations into the nitric oxide release properties of compounds **2** and **3** are also described.
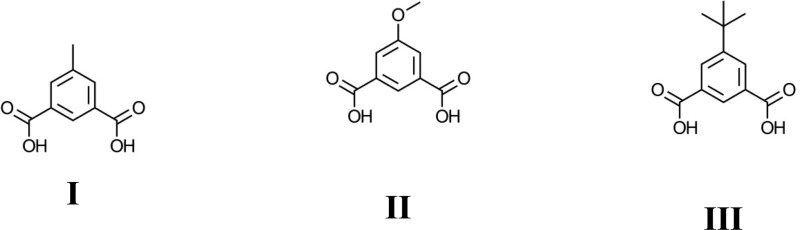


## Experimental

All reagents were obtained from commercial sources and were used without further purification.

### Syntheses

#### 5-Methoxy isophthalic acid

5-Methoxy isophthalic acid was prepared using the literature method.^18^ 5-Hydroxy isophthalic acid (18.2 g, 0.10 mol), K_2_CO_3_ (40.73 g, 0.34 mol) and dimethyl sulphate (52 mL, 0.55 mol) were heated to reflux for 18 hours in acetone (300 mL). After cooling to room temperature, the mixture was poured onto water (approx. 1.3 L). The resulting dimethyl 5-methoxyisophthalate was collected on a glass frit and recrystallised from cyclohexane. Yield: 17.47 g, 0.078 mol, 78% yield. Aqueous potassium hydroxide (5.72 M, 40 mL, 0.23 mol) was added to a methanolic solution (150 mL) of dimethyl 5-methoxyisophthalate (15.35 g, 0.068 mol) and heated to reflux for 7 hours. The cooled mixture was then poured onto distilled water (375 mL), and the solution acidified to pH 1 using conc. HCl. The crude 5-methoxy isophthalic acid was recrystallised from acetone. Yield: 12.87 g, 0.065 mol, 84%. ^1^H NMR (400 MHz, *d*_4_-MeOD) *δ* 3.91 (s, 3H, –OCH_3_), 3.95 (s, 6H, –C(O)OCH_3_), 7.75 (d, 2H, aromatic CH, *J* = 1.44 Hz), 8.20 (t, 1H, aromatic CH, *J* = 1.46 Hz) ppm. ^13^C NMR (101 MHz, *d*_4_-MeOH) *δ* 51.55, 54.88, 118.66, 122.13, 131.73, 159.94, 165.96 ppm.

#### Ni_2_(mip)_2_(H_2_O)_8_·2H_2_O (**1**)

5-Methyl isophthalic acid (108 mg, 0.60 mmol) and nickel acetate tetrahydrate (149 mg, 0.60 mmol) were suspended in a mixture of *iso*propanol (6 mL) and water (3 mL) in a Teflon-lined steel autoclave. The autoclave was heated to 110 °C for three days. During this time, the liquid in the autoclave evaporated to give green crystals of Ni_2_(mip)_2_(H_2_O)_8_·2H_2_O. Yield: 174 mg, 0.27 mmol, 89% yield. Elemental analysis calcd for Ni_2_(mip)_2_(H_2_O)_8_·2H_2_O (Ni_2_C_18_H_32_O_18_) C: 33.07, H: 4.93; found C: 33.21, H: 4.97%. IR (ATR) 3228 (br), 1613, 1606, 1534, 1420, 1365, 1248, 1117, 1042, 959, 896, 813, 773, 720 cm^–1^.

#### Zn_6_(mip)_5_(OH)_2_(H_2_O)_4_·7.4H_2_O (**2**)

5-Methyl isophthalic acid (180 mg, 1.00 mmol) and zinc acetate dihydrate (220 mg, 1.00 mmol) were suspended in a mixture of methanol (10 mL) and water (3 mL) in a Teflon-lined steel autoclave. The autoclave was heated to 110 °C for three days. Pale yellow crystals were obtained after cooling to room temperature. Yield: 205 mg, 0.14 mmol, 82% yield. Elemental analysis calcd for Zn_6_(mip)_5_(OH)_2_(H_2_O)_4_·11H_2_O (Zn_6_C_45_H_62_O_37_) C: 34.05, H: 3.94; found C: 34.01, H: 3.72%. IR (ATR) 3381 (br), 1613, 1556, 1422, 1362, 1247, 1117, 1041, 1005, 896, 810, 772, 721 cm^–1^.

#### Zn_6_(mip)_5_(OH)_2_(H_2_O)_2_·4H_2_O (**3**)

Prepared using the above procedure, with ethanol (10 mL) replacing methanol in the solvent mixture. Colourless crystals were obtained from the cooled solution. Yield: 190 mg, 0.13 mmol, 80% yield. Elemental analysis calcd for Zn_6_(mip)_5_(OH)_2_(H_2_O)_2_·4H_2_O (Zn_6_C_45_H_48_O_30_) C: 36.99, H: 3.31; found C: 37.20, H: 3.22%. IR (ATR) 3404 (br), 1617, 1541, 1430, 1371, 1317, 1305, 1253, 1240, 1118, 951, 934, 893, 806, 788, 768, 736, 723, 675, 619 cm^–1^.

#### Mn(HMeOip)_2_ (**4**)

A mixture of 5-methoxy isophthalic acid (117 mg, 0.60 mmol) and Mn(OAc)_2_·4H_2_O (147 mg, 0.60 mmol) in water (9 mL) was heated to 110 °C in a Teflon-lined steel autoclave for three days. Upon cooling to room temperature, colourless prism crystals formed. Yield: 25 mg, 60.5 μmol, 20% yield. Elemental analysis calcd for Mn(HMeOip)_2_ (MnC_18_H_14_O_10_) C: 48.56, H: 3.17; found C: 48.42, H: 3.25%. IR (ATR) 3098, 2937, 2835, 1669, 1601, 1545, 1472, 14 571 424, 1378, 1363, 1337, 1297, 1271, 1261, 1185, 1136, 1100, 1061, 928, 905, 892, 873, 798, 765, 751, 702, 684, 666, 630, 575, 546, 524 cm^–1^.

#### Mn_3_(tbip)_2_(Htbip)_2_(EtOH)_2_ (**5**)

5-*tert*-Butyl isophthalic acid (222 mg, 1.00 mmol) and Mn(OAc)_2_·4H_2_O (246 mg, 1.00 mmol) were suspended in water (5 mL) and ethanol (10 mL), and heated to 110 °C in a Teflon-lined steel autoclave for three days. Colourless plate crystals formed upon cooling to room temperature. Yield: 184 mg, 60% yield. Elemental analysis calcd for Mn_3_(tbip)_2_(Htbip)_2_(EtOH)_2_ (Mn_3_C_52_H_62_O_18_) C: 54.79 H: 5.48, found C: 54.63 H: 5.52%. IR (ATR) 2963, 1701, 1685, 1603, 1573, 1539, 1433, 1382, 1308, 1270, 1204, 1182, 1118, 1023, 948, 911, 829, 782, 772, 755, 740, 726, 702 cm^–1^.

### Crystallography

Crystals were coated in protective oil, prior to being mounted on a MiTeGen fibre tip (compounds **1**, **2** and **5**) or a Molecular Dimensions mounted 0.1 mm litholoop (compounds **3** and **4**). Single crystal data were collected on a Rigaku Mercury2 SCXMini diffractor (*λ* = 0.71075 Å, compounds **3** and **4**), or on a Bruker APEXII diffractometer (*λ* = 0.77490 Å, compounds **1**, **2** and **5**) on station 11.3.1 at the Advanced Light Source. Powder X-ray diffraction patterns were collected using Cu Kα_1_ radiation on a PANalytical Empyrean diffractometer. For data collected at beamline 11.3.1, appropriate scattering factors were applied using XDISP.^19^ Absorption corrections were applied using multi-scan methods.^20^ Structure solutions were obtained using either SHELXS-97 or SHELXT (beta) and refined by full matrix on *F* ^2^ using SHELXL-97 and SHELX-2014^21^ within the WinGX suite.^22^ All full occupancy non-hydrogen atoms were refined with anisotropic thermal displacement parameters. Aromatic and aliphatic hydrogen atoms were included at their geometrically estimated positions, as were hydrogen atoms bound to μ_3_-OH^–^ anions and non-coordinated carboxylate oxygen atoms, provided they could be located. Hydrogen atoms belonging to free and coordinated water molecules were fixed at a distance of 0.90 Å from the oxygen atom and 1.47 Å from the other hydrogen bound to the same oxygen atom, and their thermal displacement parameters linked to that of the oxygen to which they are bound.

### Nitric oxide loading and release

Powders were weighed into individual ampules, which were then placed in a Schlenk flask. The flask was heated to 200 °C and held at this temperature under dynamic vacuum for approximately 18 hours. After this time, the flask was cooled to room temperature and filled with NO (∼1 atm static pressure) for 1 hour. Excess NO was then removed by evacuating the flask and flushing with Ar three times. The ampules were then individually flame-sealed under an argon atmosphere. NO release measurements were performed using a Sievers NOA 280i chemiluminescence Nitric Oxide Analyser. The instrument was calibrated using air passed through a zero filter (<1 ppb NO) and 87.6 ppm NO gas in N_2_ (Air Products). The flow rate through the instrument was set to 200 mL min^–1^ with a cell pressure of approximately 5.2 Torr and an oxygen pressure of 6.0 psig. Nitrogen gas of known humidity was achieved by passing dry N_2_ over a saturated aqueous solution of LiCl to give 11% relative humidity. Humid N_2_ was passed over the powders at room temperature, and the resulting NO/N_2_ mixture was then passed through the analyser and the NO concentration recorded at 1 second intervals. Data collection was stopped after the concentration of NO in the carrier gas dropped below 20 ppb.

## Results and discussion

All five compounds were prepared by solvothermal reaction between metal acetate and 5-substituted isophthalic acid in either water or aqueous alcohol. A summary of the crystallographic details is presented in Table 1. Compounds **1** and **2** crystallise in polar space groups and their Flack parameters indicate that these crystals are twinned by inversion.

**Table 1:** Crystallographic details for the structure determinations of compounds **1** to **5**

Compound	**1**	**2**	**3**	**4**	**5**
Empirical formula	C_18_H_32_Ni_2_O_18_	C_45_H_54.75_O_33.5_Zn_6_	C_45_H_44_O_28_Zn_6_	C_18_H_14_MnO_10_	C_52_H_62_Mn_3_O_18_
MW	653.85	1523.85	1425.02	445.23	1139.83
*λ* (Å)	0.77490	0.77490	0.71075	0.71075	0.77490
*T* (K)	150(2)	150(2)	173(2)	173(2)	150(2)
Crystal system	Orthorhombic	Orthorhombic	Orthorhombic	Monoclinic	Triclinic
Space group	*Pna*2_1_	*Pnn*2	*P*2_1_2_1_2	*P*2_1_/*c*	*P*1
*a* (Å)	15.4414(15)	20.775(3)	17.382(4)	4.5131(8)	9.1496(12)
*b* (Å)	10.9156(11)	27.566(3)	10.4516(15)	14.483(3)	9.7908(12)
*c* (Å)	14.5960(14)	10.8460(13)	15.970(3)	12.622(3)	16.078(2)
*α* (°)	90	90	90	90	89.207(2)
*β* (°)	90	90	90	97.792(9)	76.087(2)
*γ* (°)	90	90	90	90	74.643(2)
*V* (Å^3^)	2460.2(4)	6211.4(13)	2901.3(10)	817.4(3)	1346.2(3)
*Z*	4	4	2	2	1
Flack parameter	0.481(14)	0.491(4)	0.01(3)	—	—
*ρ*_calcd_ (g cm^–1^)	1.765	1.630	1.631	1.809	1.406
*μ* (mm^–1^)	2.040	2.992	2.524	0.871	0.960
*F*(000)	1360	3091	1436	454	593
GoF	1.085	1.042	1.073	1.032	1.041
Reflections collected/unique	34 124/7435	90 099/18 933	30 286/6666	8512/1877	20 014/8056
No. of parameters	397	767	358	135	343
*R*_int_	0.0613	0.0476	0.1248	0.0563	0.0538
Final *R* indices *I* > 2*σ*(*I*)	*R*_1_ = 0.0377 w*R*_2_ = 0.1051	*R*_1_ = 0.0533 w*R*_2_ = 0.1378	*R*_1_ = 0.0575 w*R*_2_ = 0.1285	*R*_1_ = 0.0348 w*R*_2_ = 0.0737	*R*_1_ = 0.0453 w*R*_2_ = 0.1204
*R* indices (all data)	*R*_1_ = 0.0399 w*R*_2_ = 0.1068	*R*_1_ = 0.0618 w*R*_2_ = 0.1432	*R*_1_ = 0.1102 w*R*_2_ = 0.1517	*R*_1_ = 0.0593 w*R*_2_ = 0.0829	*R*_1_ = 0.0538 w*R*_2_ = 0.1260

**Fig. 1:**
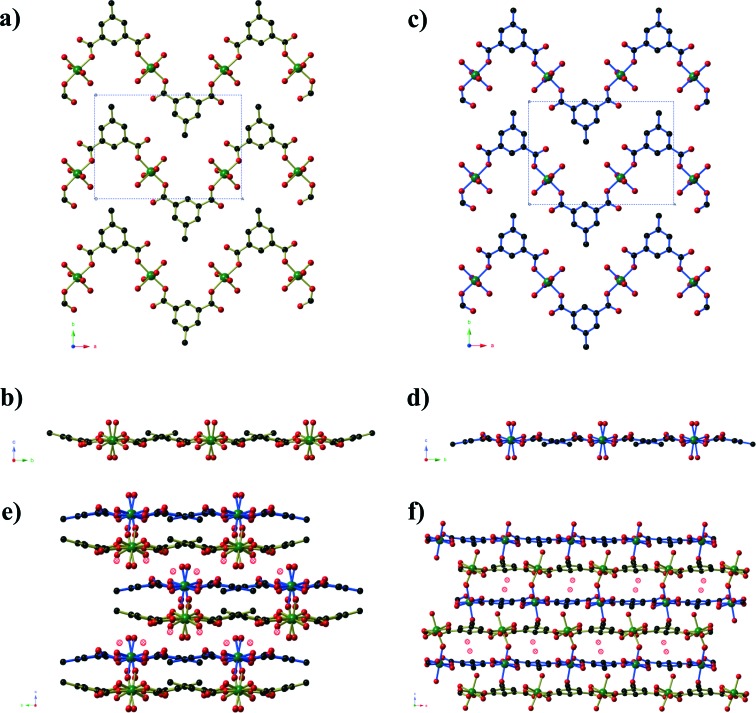
The crystal structure of Ni_2_(mip)_2_(H_2_O)_8_·2H_2_O (compound **1**). One layer of Type 1 Ni(mip)(H_2_O)_4_ chains as seen **a)** perpendicular and **b)** parallel to the plane of the layer. One layer of Type 2 Ni(mip)(H_2_O)_4_ chains as seen **c)** perpendicular and **d)** parallel to the plane of the layer. **e)** The stacking of pairs of layers along the *c*-axis as viewed along the *a*-axis. Non-coordinated water molecules are shown as hatched red spheres. **f)** Stacking of the layers as seen parallel to the *b*-axis. Hydrogen atoms have been omitted for clarity.

Compound **1**, Ni_2_(mip)_2_(H_2_O)_8_·2H_2_O, crystallises in the polar space group *Pna*2_1_, and the asymmetric unit contains two crystallographically distinct nickel centres, two 5-methyl isophthalate (mip) ligands and ten water molecules. Both nickel centres are bound to two *trans* symmetry-related mip ligands (Fig. 1) and four water molecules, leading to two distinct chains (Types 1 and 2) that extend parallel to the *a*-axis. The methyl isophthalate ligands do not lie in the average plane of the chain, but rather are angled such that all methyl groups on a given chain lie on one face of the chain, giving each chain a very slightly U-shaped cross section. Symmetry-related chains are arranged into layers that lie in the *ab* plane. Within each layer, the U-shaped chains are arranged such that all of the methyl groups lie above the same face of the layer. As seen in Fig. 1c, the methyl groups of Type 1 chains all lie on the upper face of the layer whereas the methyl groups of Type 2 layers lie on the lower face of the layer. Pairs of layers form, consisting of one Type 1 and one Type 2, in which the methyl groups of both layers are directed towards the centre of the pair. These pairs of layers then stack parallel to the *c*-axis in an ABAB fashion. Extensive hydrogen bonds between the coordinated water molecules and carboxylate oxygen atoms of chains belonging to different layers connect the chains into a complex three-dimensional hydrogen-bonded framework. Water molecules of crystallisation occupy the spaces between adjacent pairs of layers and are held in place by hydrogen bonds to the chains.

**Fig. 2:**
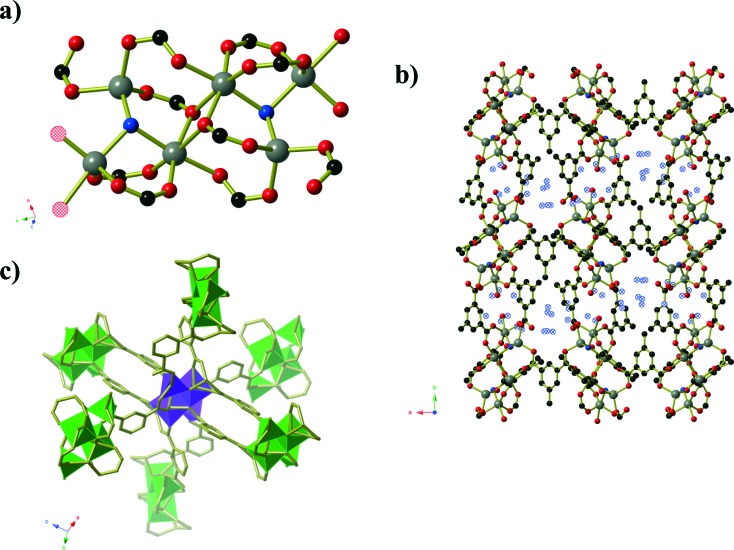
**a)** The Zn_6_(OH)_2_(CO_2_)_10_(H_2_O)_3_ cluster found in Zn_6_(mip)_5_(OH)_2_(H_2_O)_4_·*x*H_2_O (*x* ≈ 7.4, compound **2**). The two μ_3_-OH anions are shown in blue, and major sites of the disordered coordinated water molecules are shown as hatched red circles. **b)** A view along the channels of the α-Po net of Zn_6_(mip)_5_(OH)_2_(H_2_O)_4_·*x*H_2_O (*x* ≈ 7.4). Partial occupancy water molecules are shown as hatched blue spheres. Hydrogen atoms have been omitted for clarity. **c)** The connectivity of the Zn_6_(OH)_2_(CO_2_)_10_(H_2_O)_3_ clusters which act as six-connecting nodes in the α-Po net. The Zn centres belonging to the central and peripheral clusters are shown in purple and green, respectively. Terminal methyl groups, coordinated water molecules and monodentate carboxylate groups have been omitted for clarity.

Compounds **2** and **3** are formed by reaction of zinc acetate and 5-methyl isophthalic acid, differing only in which organic solvent is used in conjunction with water. The two frameworks obtained, however, are vastly different to one another and are distinct from the five literature coordination frameworks^23^ and metal–organic polyhedra^24^ that are also composed of zinc(ii) and 5-methyl isophthalate in the absence of additional metal ions or bridging ligands. Compound **2**, produced from aqueous methanol, has composition Zn_6_(mip)_5_(OH)_2_(H_2_O)_4_·*x*H_2_O (*x* ≈ 7. 4) forms in orthorhombic space group *Pnn*2. The asymmetric unit contains six crystallographically distinct Zn^II^ centres, 5 distinct mip ligands, 2 μ_3_-OH^–^ anions, three coordinated water molecules and several disordered guest water molecules. There is some rotational disorder of the mip ligands, hinged about the two carboxylate groups, and the coordinated water molecules. The Zn^II^ centres are arranged to form clusters of composition Zn_6_(OH)_2_(CO_2_)_10_(H_2_O)_3_ (Fig. 2). The clusters act as a 6-connecting node, binding to four symmetry-related clusters in the *bc*-plane *via* pairs of bridging mip ligands and to two symmetry-related clusters above and below this plane *via* a single mip ligand. The resulting three-dimensional coordination framework has the α-polonium topology. Narrow channels (approx. 4 × 6 Å) extend parallel to the *c*-axis that are bordered on one ‘end’ by the methyl groups of the mip ligand and on the other by carboxylate groups. The hydrophobic section of the channels is completely blocked by the ligands; however, disordered water molecules occupy the hydrophilic section. Disordered water molecules also occupy the smaller channels (3 × 4.5 Å) that lie between the clusters.

**Fig. 3:**
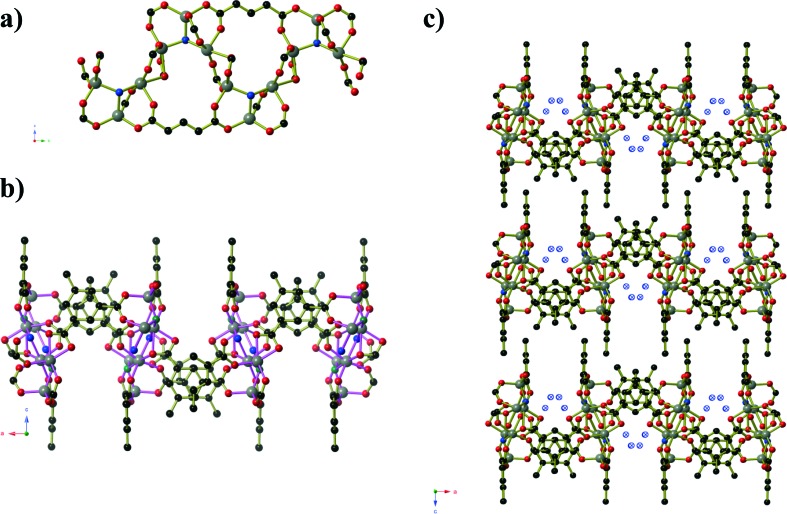
**a)** A view of the columns of Zn_3_(OH) clusters in Zn_6_(mip)_5_(OH)_2_(H_2_O)_2_·*x*H_2_O (*x* = 4, compound **3**). **b)** One sheet of Zn_6_(mip)_5_(OH)_2_(H_2_O)_2_·*x*H_2_O (*x* = 4) as seen from along the plane of the sheet. The columns from Fig. 3a are highlighted using pink Zn–O bonds and extend into the page. **c)** The packing of the sheets of Zn_6_(mip)_5_(OH)_2_(H_2_O)_2_·*x*H_2_O (*x* = 4). The μ_3_-OH anions are shown as blue spheres, whilst guest water molecules are shown as hatched blue spheres.

Compound **3**, formed from aqueous ethanol, also has composition Zn_6_(mip)_5_(OH)_2_(H_2_O)_2_·*x*H_2_O (*x* = 4) and crystallises in orthorhombic space group *P*2_1_2_1_2. The asymmetric unit contains three crystallographically distinct Zn^II^ centres, two and a half mip ligands, one μ_3_-OH, one coordinated water molecule and two full occupancy guest water molecules. Unlike in compound **2**, the Zn centres in compound **3** are arranged into Zn_3_(OH) units (Fig. 3) that are connected into ‘columns’ by μ_2_ carboxylate oxygen atoms and Type 3 mip ligands. The remaining two mip ligands connect these ‘columns’ into corrugated sheets that extend parallel to the *ab*-plane, in which the Types 1 and 2 mip ligands form the peaks and troughs of the sheet and the methyl groups of Type 3 mip ligands project outward from the plane of the sheet on either side of the peaks and troughs. The Type 1 and 2 mip ligands alternate parallel to the *b*-axis, where each Type 1 ligand is involved in face-to-face aromatic interactions with two Type 2 ligands, and *vice versa*, with close contact C···C distances of 3.4 to 3.8 Å. Sheets stack along the *c*-axis, with the methyl groups of Type 3 mip ligands interdigitating with those belonging to adjacent sheets. Water molecules occupy the ‘channels’ between the corrugated sheets.

**Fig. 4:**
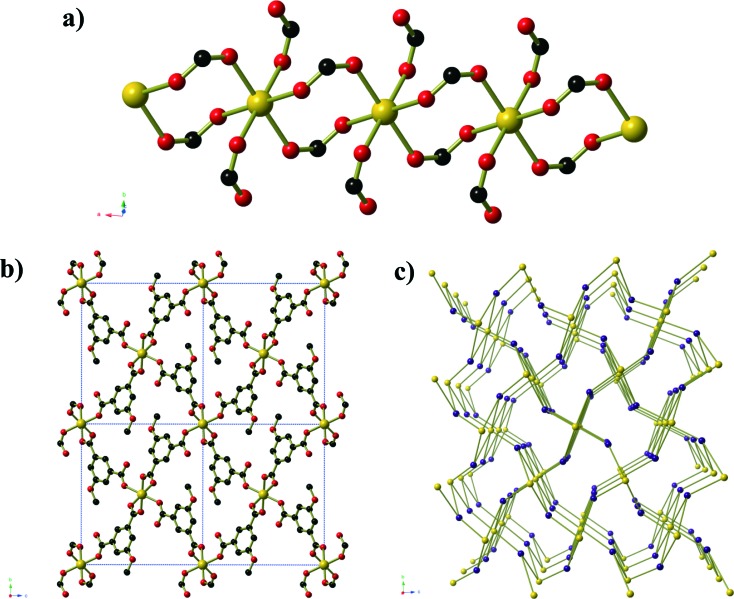
**a)** Chains of carboxylate bridged Mn^II^ centres in Mn(HMeOip)_2_ (compound **4**). **b)** The full Mn(HMeOip)_2_ framework. **c)** A schematic representation of the rutile topology, in which the three-connecting HMeOip^–^ ligands are represented by purple spheres. Hydrogen atoms have been omitted for clarity.

Compound **4** forms as a three-dimensional coordination polymer of composition Mn(HMeOip)_2_ in space group *P*2_1_/*c*. One Mn^II^ centre and one HMeOip^–^ monoanion make up the asymmetric unit. The HMeOip^–^ ligand acts as a 3-connecting node, binding to one metal centre through the carbonyl oxygen of the carboxylic acid, and the carboxylate group binds to a further two, one per oxygen atom (Fig. 4). The Mn^II^ centre adopts an octahedral coordination environment in which four bridging carboxylate groups are arranged in a square plane whilst the two monodentate carboxylic acid groups bind in a *trans* arrangement. Bridging carboxylate groups connect the Mn centres into chains of Mn(CO_2_)_2_Mn that extend parallel to the *a*-axis. These chains are then cross-linked by the coordinated carboxylic acid to afford a three-dimensional network with rutile topology. Intra-chain hydrogen bonds (O···O separation of 2.657(3) Å) occur between the carboxylic OH group and one oxygen atoms of the bridging carboxylate group.

**Fig. 5:**
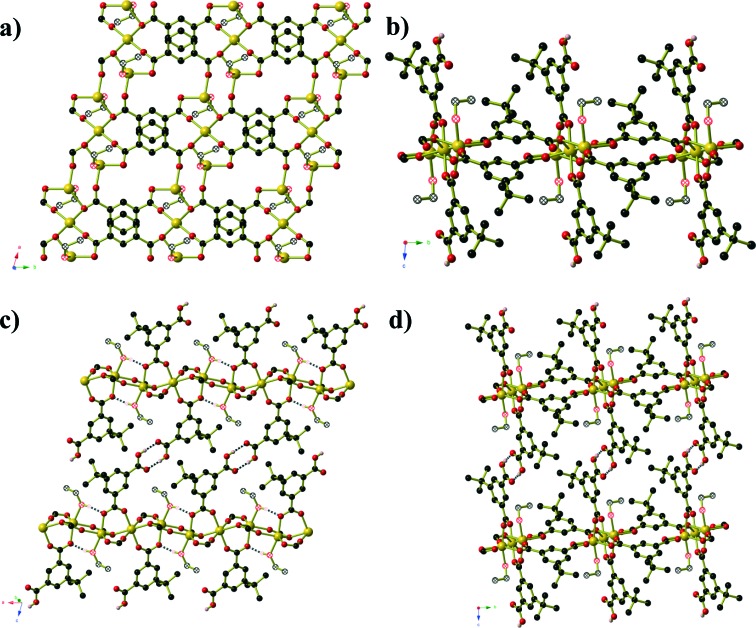
**a)** One sheet of Mn_3_(tbip)_2_(Htbip)_2_(EtOH)_2_ (compound **5**) as viewed perpendicular to the plane of the sheet. The atoms of the coordinated ethanol molecules are shown using hatched spheres. The Htbip^–^ ligands have been omitted for clarity. **b)** View along the edge of a complete sheet. **c)** The hydrogen-bonding interactions between adjacent sheets. Hydrogen bonds are shown as black and white striped bonds. **d)** The packing of the sheets along the *c*-axis.

Compound **5** is analogous to the literature compound Mn_3_(tbip)_2_(Htbip)_2_(^i^PrOH)_2_ with ethanol replacing the coordinated isopropanol molecules.^25^ The compound forms in space group *P*1 and the asymmetric unit contains two Mn(ii) centres, a coordinated ethanol molecule, one tbip^2–^ ligand and one Htbip^–^ ligand. The fully deprotonated tbip^2–^ units connect the Mn centres into a two dimensional sheet (Fig. 5) that extends parallel to the *ab*-plane. Within this sheet, the Mn centres are grouped into trios containing one central octahedral Mn centre and two peripheral trigonal bipyramidal Mn centres that are connected *via* μ_2_ carboxylate oxygen atoms and bridging carboxylate groups that lie in the basal planes of the three Mn centres. These trios are connected by bridging carboxylate groups (also binding in the basal planes of the Mn centres) into chains running parallel to the [1 0 0] direction. Pairs of tbip^2–^ ligands connect these chains together, oriented at an angle of approximately 34° to and sitting slightly above and below the plane of the sheet. These pairs of ligands are arranged such that they form a Piedfort unit with close contact C···C separations of 3.67 to 3.80 Å. The axial coordination sites on the octahedral Mn centres are occupied by the deprotonated carboxylate group of the Htbip^–^ ligands, the second oxygen atoms of which binds in one axial site of an adjacent trigonal bipyramidal Mn centre. A coordinated ethanol molecule occupies the remaining coordination site on the trigonal bipyramidal Mn centres and forms an intramolecular hydrogen bond to the coordinated carboxylate oxygen atom on the adjacent octahedral Mn centre. The carboxylic acid groups of the Htbip^–^ ligands project outwards from the plane of the sheet, where they form complementary pairs of hydrogen bonds with symmetry-related ligands on adjacent sheets. These hydrogen bonds connect the 2D framework of Mn_3_(tbip)_2_(Htbip)_2_(EtOH)_2_ into a three-dimensional hydrogen-bonded network.

## Nitric oxide release

Preliminary screening was conducted on compounds **2** and **3** for their nitric oxide release capacity. These compounds were selected because they contained coordinated solvent molecules that may be removed to generate a vacant coordination site whilst potentially leaving the coordination framework intact. compound **5** was not considered as removal of the coordinated ethanol molecules would most likely disrupt the inter-sheet hydrogen bonds. Thermogravimetric analyses were conducted on all 5 compounds, and are presented in ESI.† Samples were dehydrated at 200 °C under dynamic vacuum for approximately 18 hours, before being exposed to a nitric oxide atmosphere for approximately 1 hour. The samples were flame-sealed in an argon atmosphere. Nitric oxide release was triggered by exposure to humid N_2_ (∼11% RH). Initial measurements showed that the NO release from these compounds was very time sensitive, so repeat measurements were conducted in two phases – as soon as possible after NO loading, and then after 1 week (Tables 2 and 3). The NO-release from compound **3** proved to be erratic. Both samples showed a small initial release on the day of loading, in the range of 0.16–0.21 μmol g^–1^ for compound **2** and 9.5–17.5 μmol g^–1^ for compound **3**. These values are small but occur over a period of minutes for compound **2** and hours for compound **3**, which is consistent with the loss of NO that has been included into the pores of the material rather than simply being physisorbed onto the surface of the powder. The difference in the amount of NO released from compounds **2** and **3** we attribute to the larger cross-sectional area of the channels in compound **3** compared to compound **2**, as may be seen by comparison of Fig. 2b and 3c. The channels in compound **2** have a corrugated surface and are approximately 6 Å high and 2 Å wide, whereas those in compound **3** are approximately 8.5 Å high and between 2.7 and 5.5 Å wide. The larger the cross-sectional area of the channel opening allows for easier ingress and egress of the NO and water molecules. The amount of NO released less than 1 day after loading is comparable to the release from other well-known materials such as HKUST-1 (NO release 2 μmol g^–1^) and this amount of NO has been shown to have biological effects (*e.g.* anti-platelet activation).^26^ After standing for one week, however, the amount of released NO drops by a factor of 40 for compound **2** and by two orders of magnitude for compound **3**. The release time was also significantly lower after this short storage time, signifying that the NO-loaded compounds have a very short shelf-life and these compounds would not be suitable for use as biomedical NO delivery systems.

**Table 2:** Details of NO release from compound **2**

Zn_6_(mip)_5_(OH)_2_(H_2_O)_4_·*x*H_2_O (*x* ≈ 7.4)	Pre-loaded weight (mg)	Loaded weight	Total NO released (μmol g^–1^)	Release time (min)	Peak NO release
1	15.5	13.1	0.21	4.5	2.4 ppm
2	15.2	13.0	0.18	4.5	2.0 ppm
3	15.5	13.1	0.18	4.5	1.8 ppm
4	15.1	12.8	0.16	4.5	1.5 ppm
5 (after 1 week)	15.3	13.5	0.0059	<1	81.8 ppb
6 (after 1 week)	15.4	13.3	0.0036	<1	62.8 ppb
7 (after 1 week)	15.4	12.9	0.0027	<1	43.3 ppb
8 (after 1 week)	15.5	13.1	0.0051	<1	93.2 ppb

**Table 3:** Details of NO release from compound **3**

Zn_6_(mip)_5_(OH)_2_(H_2_O)_4_·*x*H_2_O (*x* ≈ 4)	Pre-loaded weight (mg)	Loaded weight	Total NO released (μmol g^–1^)	Release time (min)	Peak NO release
1	15.1	14.2	17.10	93	12.9 ppm
2 (after 1 day)	15.1	14.6	9.87	60	2.8 ppm
3 (after 1 day)	15.3	13.9	9.72	118	3.2 ppm
4 (after 1 week)	15.3	13.8	0.46	10	1.2 ppm
5 (after 1 week)	15.2	13.8	0.98	19	1.3 ppm
6 (after 1 week)	15.0	13.6	2.75	59	1.2 ppm

**Fig. 6:**
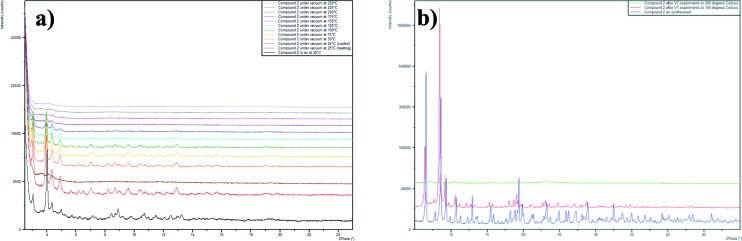
**a)** Variable temperature PXRD traces for compound **2**. **b)** PXRD patterns on compound **2** on samples that are as synthesised (blue), after VT studies to 150 °C (red) and after VT studies to 250 °C (green).

Powder diffraction patterns were collected on both compounds before and after NO-loading and release experiments. This showed that the PXRD pattern for compound **3** does not change as a result of the NO-loading and release, indicating that the framework remains intact throughout the dehydration–NO-loading–rehydration process. The PXRD pattern for compound **2** after NO release contains one extra peak at 2*θ* = 7°, which may be indicative of a contaminent being formed from the partial decomposition of compound **2**. Variable temperature PXRD studies showed that compound **2** remains largely unchanged when heated to 150 °C, after this temperature some degradation of the sample occurs (Fig. 6). Upon heating to 250 °C, the sample is completely destroyed and further powder diffraction studies showed that the residue contains a peak centred at 2*θ* = 7°.

## Conclusions

One derivative and four novel coordination polymers have been prepared by solvothermal reaction of metal acetate and 5-substituted isophthalic acid in either water or aqueous short-chain alcohols. In the case of zinc and 5-methyl isophthalate, varying the length of the carbon chain in the organic solvent can influence the connectivity and dimensionality of the resulting coordination framework. Preliminary NO release screening showed that compounds **2** and **3** are able to deliver small amounts of NO, however the shelf life of the NO-loaded compounds is limited.
