# Supplementary material for: Coordination polymers of 5-substituted isophthalic acid
Source: CrystEngComm. 2015 Dec 21;18(7):1123–32. doi: 10.1039/c5ce02091c (PMC4786948; doi:10.1039/c5ce02091c)
Supplement: Supplementary file 1 [file CE-018-C5CE02091C-s001.pdf]

Electronic Supplementary Material (ESI) for CrystEngComm.
This journal is © The Royal Society of Chemistry 2015

Coordination polymers of 5-susbstituted isophthalic acid

Laura J. McCormick,[a*] Samuel A. Morris,[a] Alexandra M. Z. Slawin,[a] Simon J. Teat[b] and Russell E. Morris[a]

[a] EaSTCHEM School of Chemistry, University of St Andrews, North Haugh, St Andrews, Fife, Scotland, KY16 9ST, UK. E-mail: ljm22@st-andrews.ac.uk; Fax: +44 (0)1334 463808; Tel: +44 (0)1334 463776

[b] Advanced Light Source, Berkeley Laboratory, 1 Cyclotron Road, Berkeley, California 94720, USA

S1 Powder diffraction patterns

S2 Thermogravimetric studies

S3 Details of Nitric Oxide release experiments

S4 Comparison of powder diffraction patterns before and after NO-loading and release

S5 Selected bond lengths and distances for Compounds **1** to **5**

S1 Powder Diffraction Patterns

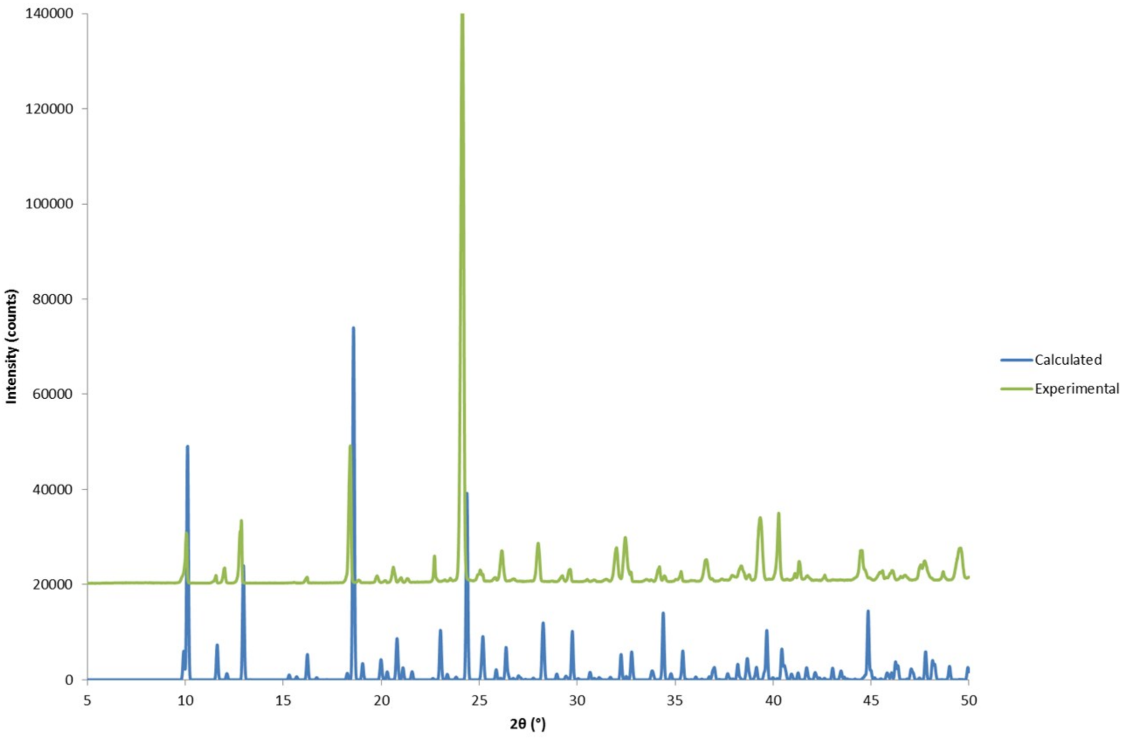

Figure S1.1: Calculated and experimental powder diffraction patterns for $Ni_2(mip)_2(H_2O)_8 \cdot 2H_2O$ (**1**).

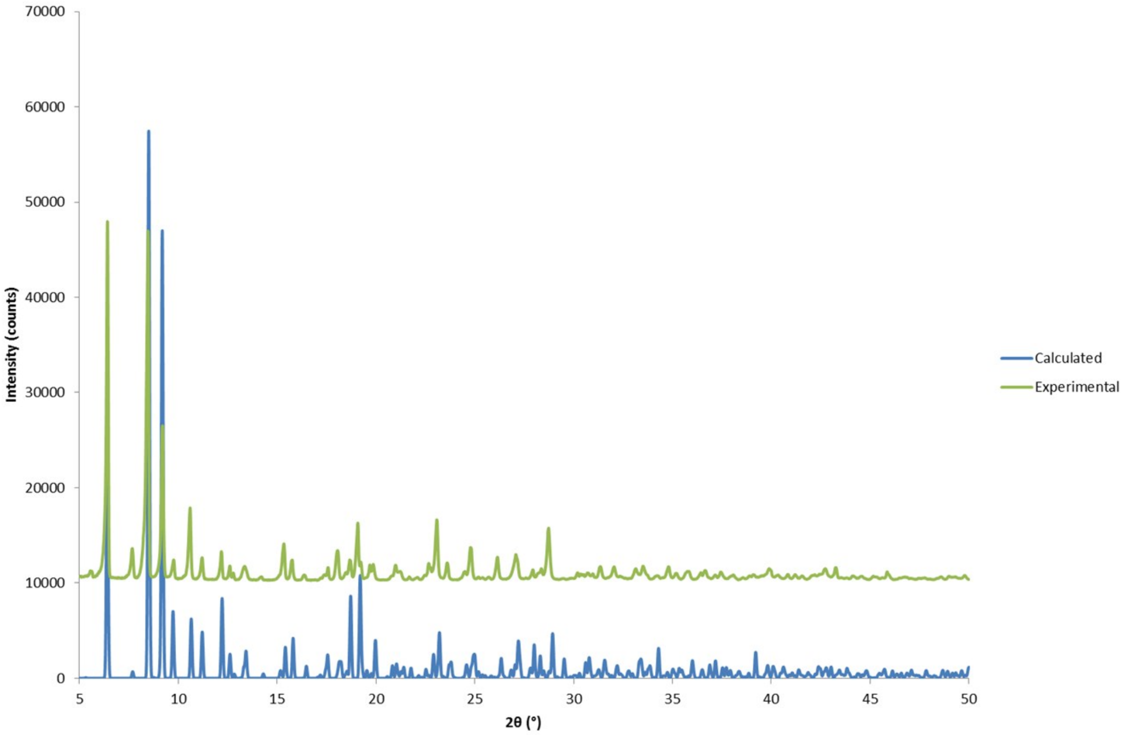

Figure S1.2: Calculated and experimental powder diffraction patterns for $Zn_6(mip)_5(OH)_2(H_2O)_4 \cdot 6.1H_2O$ (**2**).

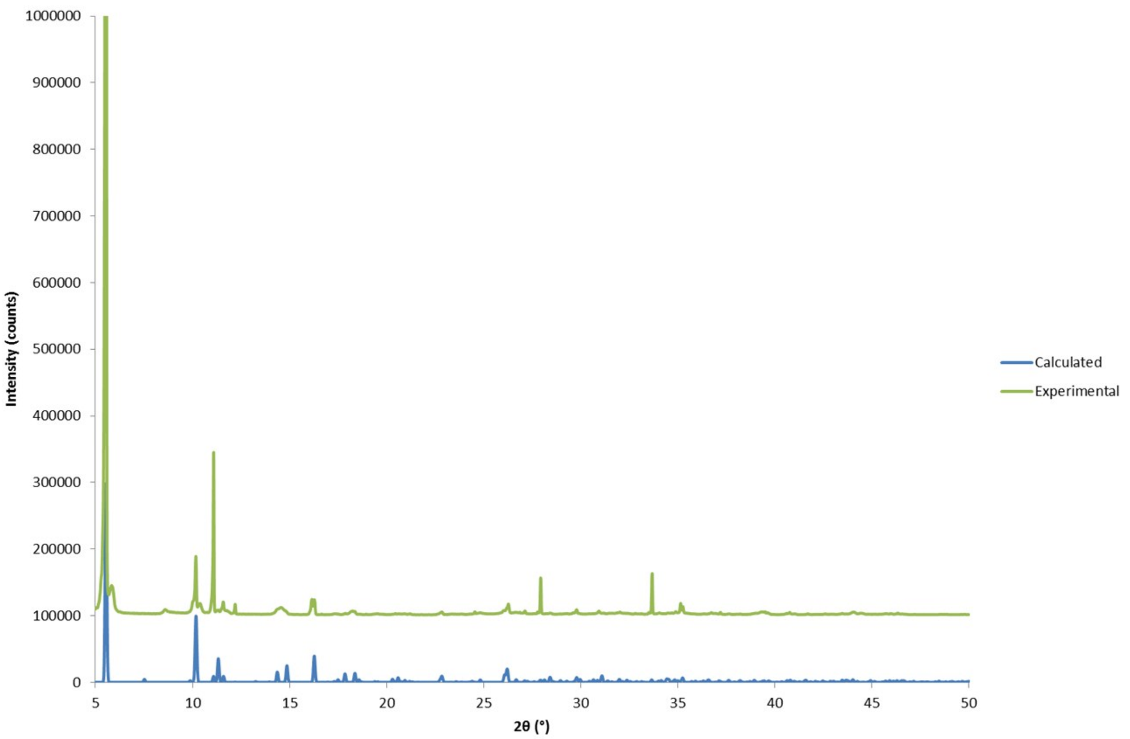

Figure S1.3: Calculated and experimental powder diffraction patterns for $Zn_6(mip)_5(OH)_2(H_2O)_2 \cdot 4H_2O$ (**3**).

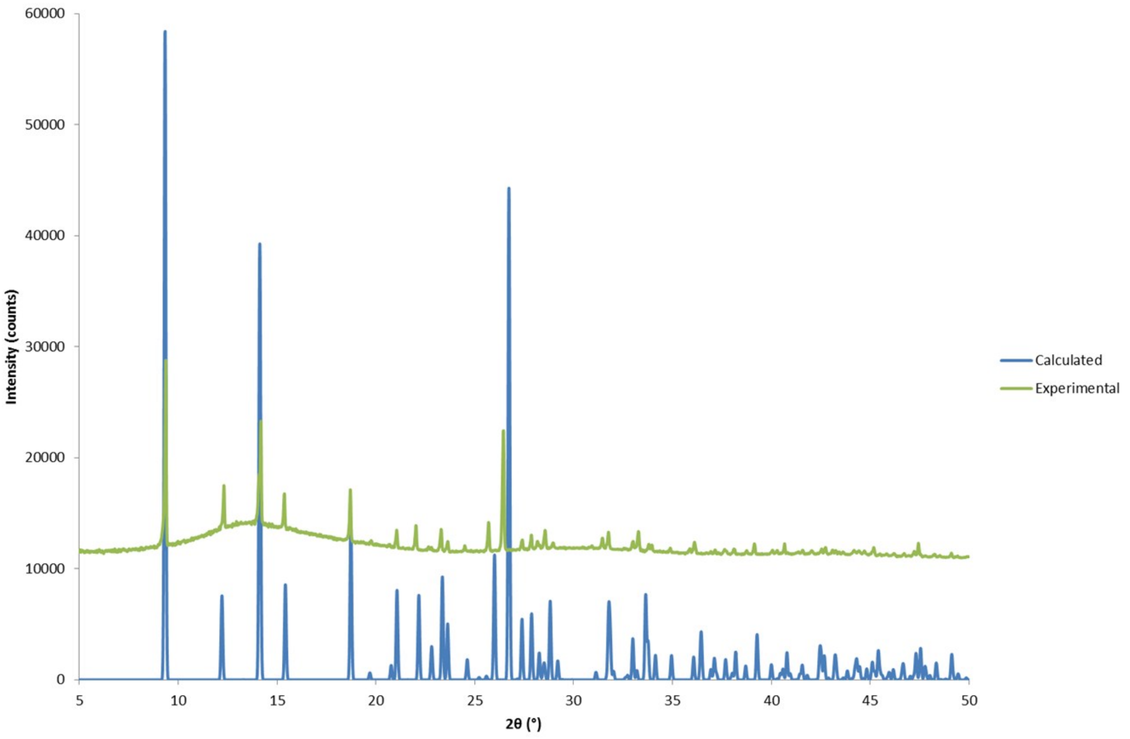

Figure S1.4: Calculated and experimental powder diffraction patterns for $Mn(HMeOip)_2$ (**4**).

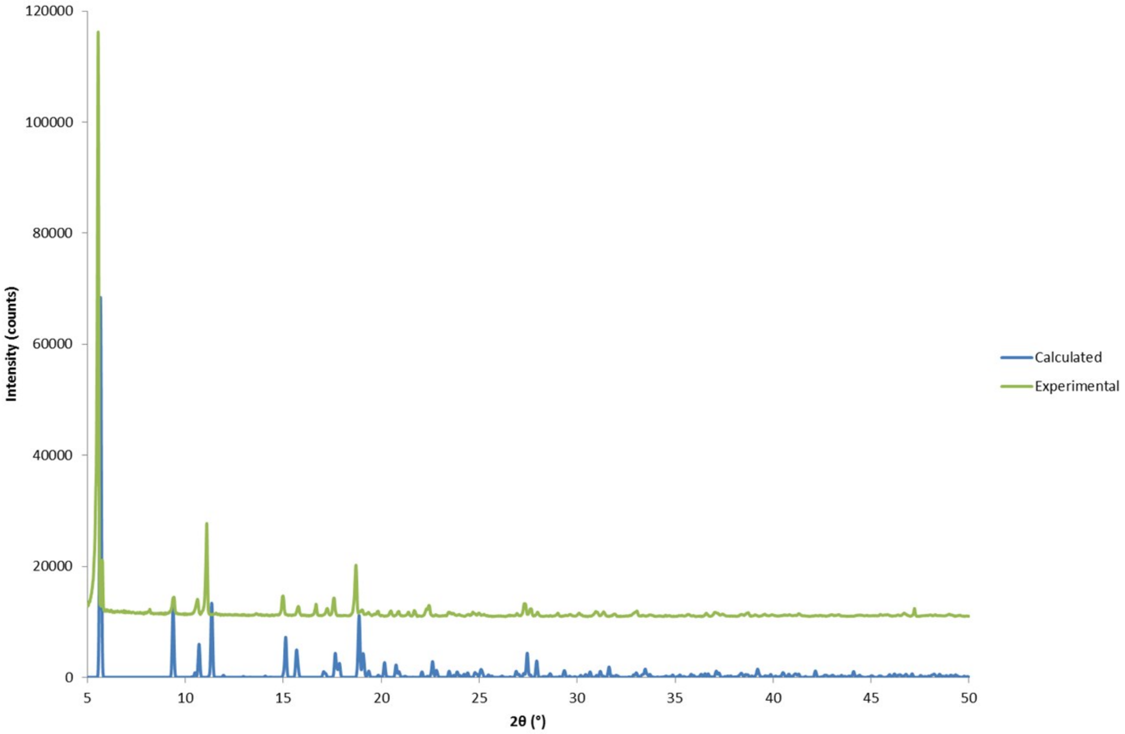

Figure S1.5: Calculated and experimental powder diffraction patterns for $Mn_3(tbip)_2(Htbip)_2(EtOH)_2$ (**5**).

S2 Thermogravimetric experiments

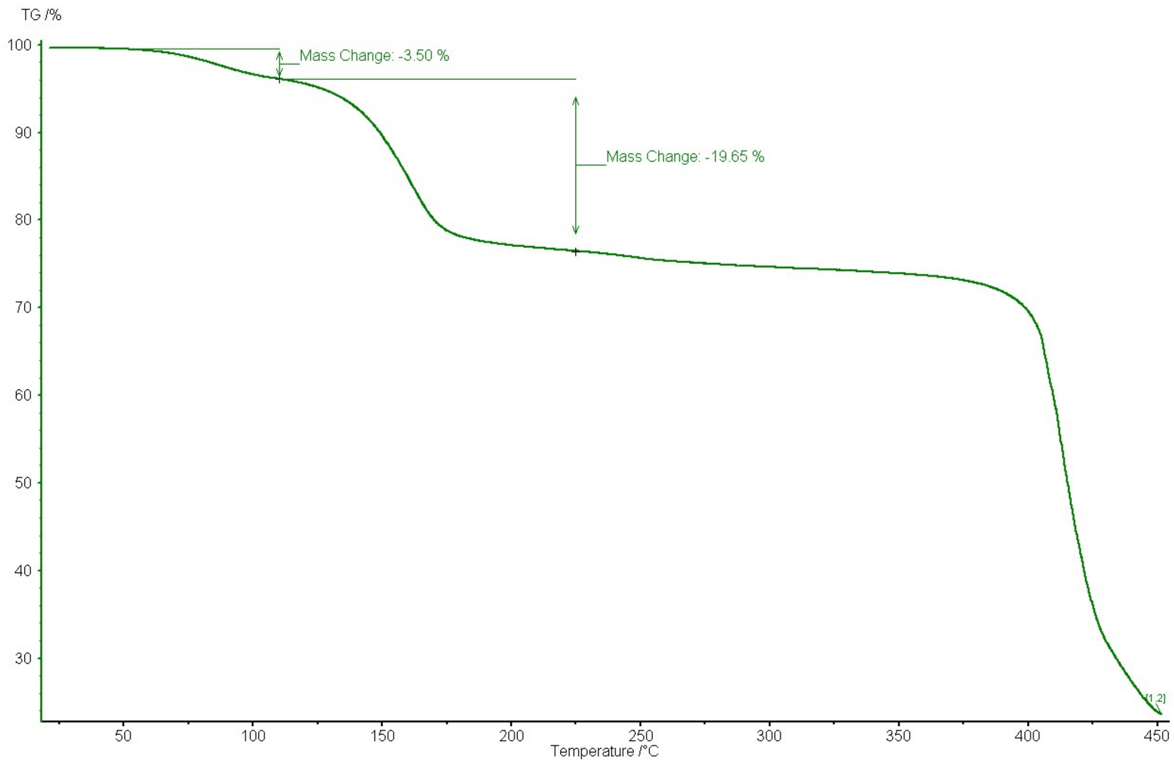

Figure S2.1: Thermogravimetric analysis of Compound **1**. Overall mass loss (23.15%) corresponds to the loss of 10.93 water molecules, of which 1.65 water molecules are lost in the first step (25 to 110°C, 3.49%, non-coordinated water molecules) and 9.28 are lost in the second step (110 to 225°C, 19.65%, coordinated water molecules).

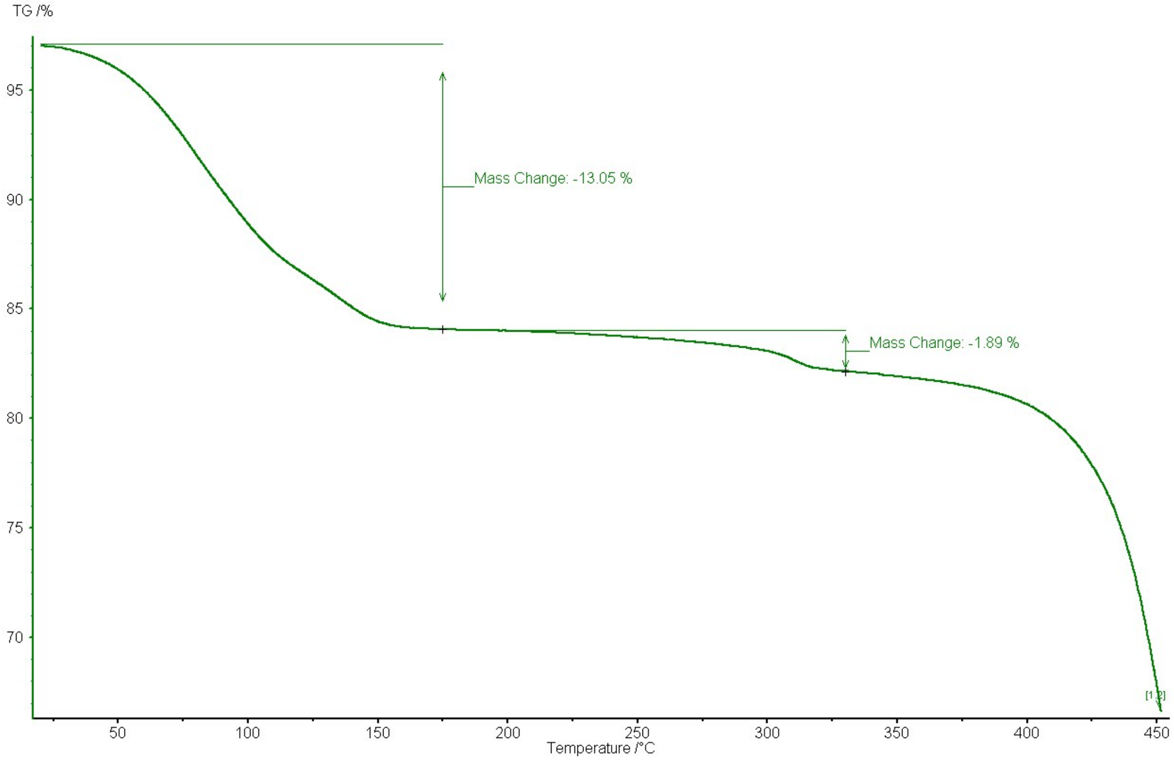

Figure S2.2: Thermogravimetric analysis of Compound **2**. Initial mass loss occurring between 20 and 175°C corresponds to the loss of approximately 11 water molecules (13.07%).

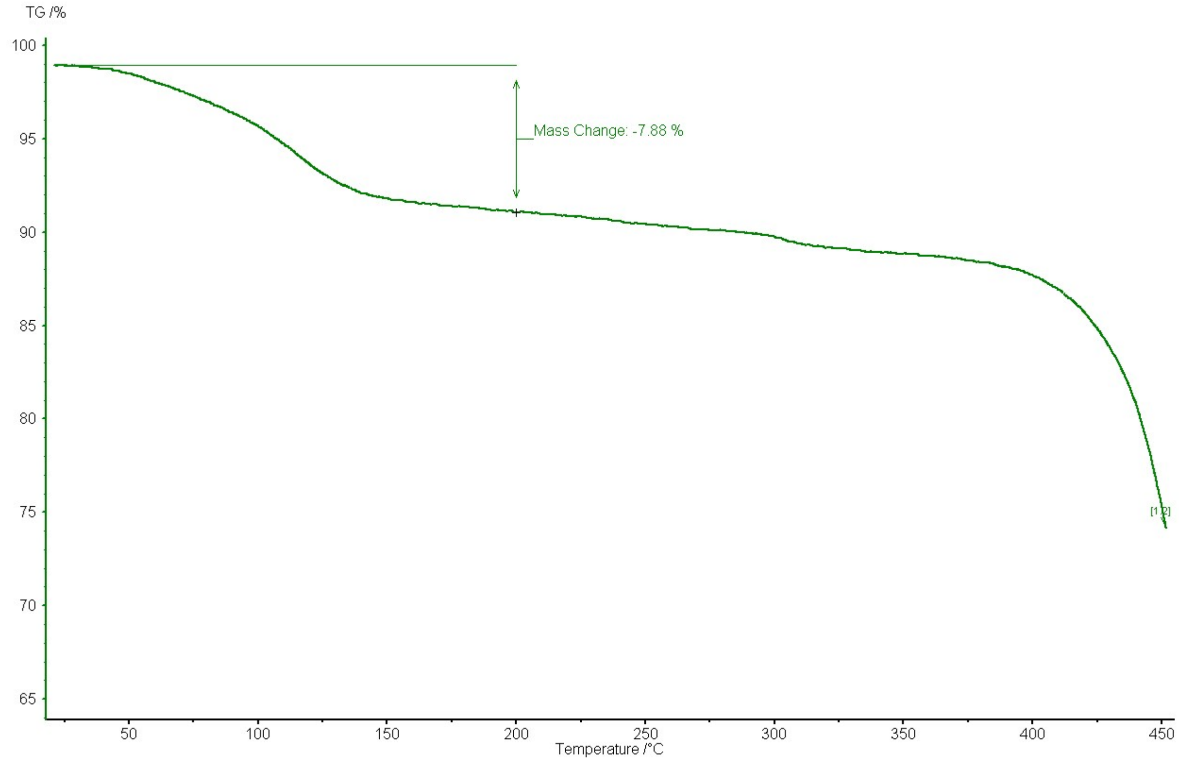

Figure S2.3: Thermogravimetric analysis of Compound **3**. The mass loss occurring over the temperature range 20°C to 200°C approximately corresponds to the loss of 6 water molecules (7.58%).

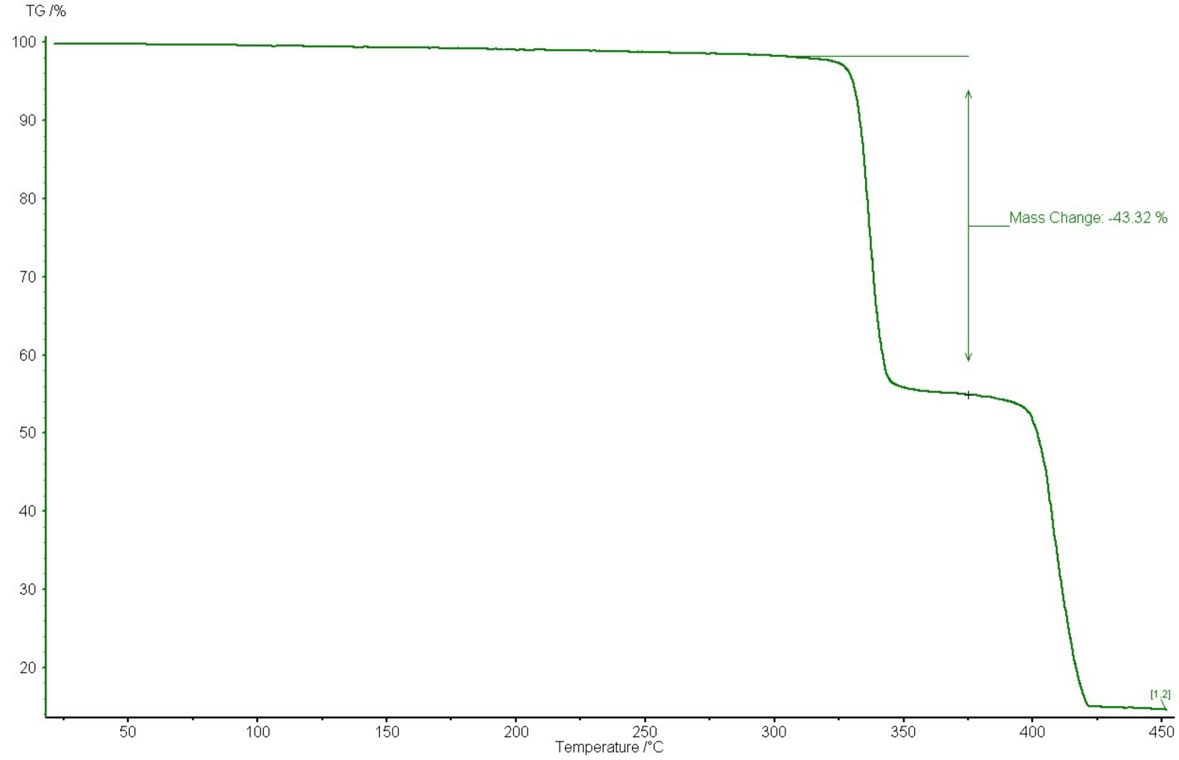

Figure S2.4: Thermogravimetric analysis of Compound **4**. The compound is stable up to approximately 300°C, at which point a mass loss occurs corresponding to more than the loss of both carboxylate groups (35.34%).

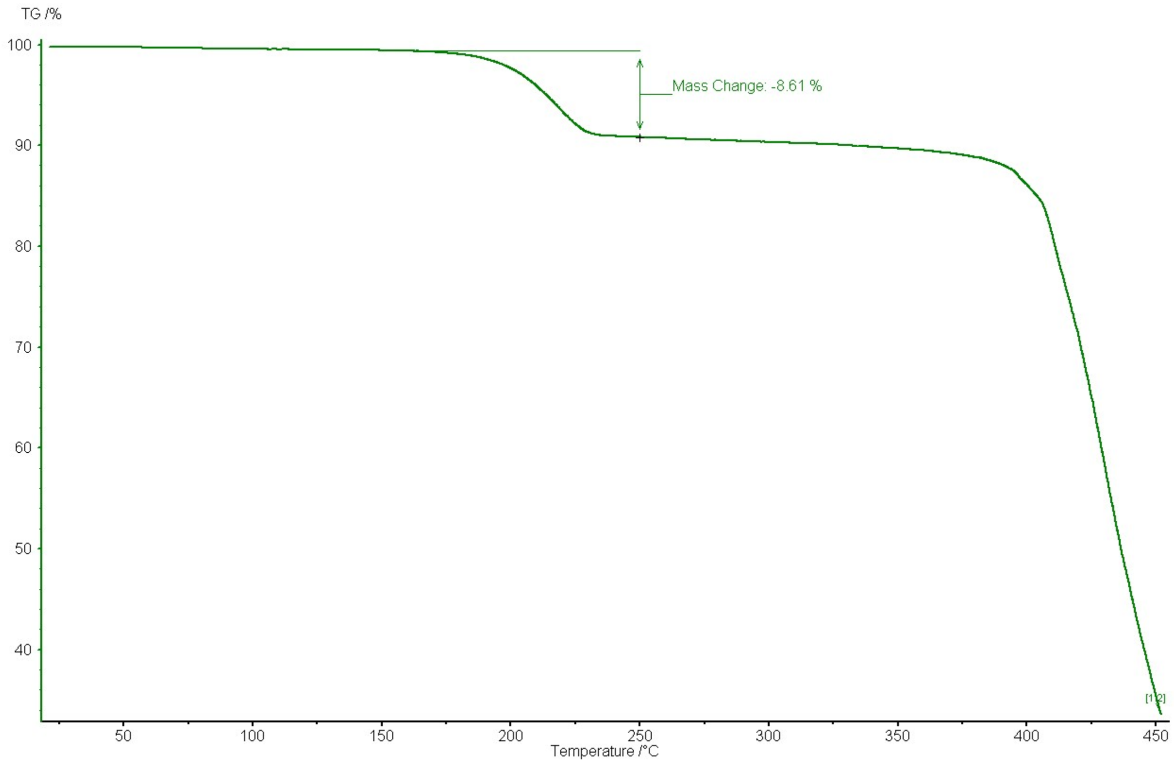

Figure S2.5: Thermogravimetric analysis of Compound **5**. The mass loss occurring over the 150° to 250°C temperature range approximately corresponds to the loss of the coordinated ethanol molecule (10.35%).

S3 Details of Nitric Oxide release experiments

*S3.1 Plots of Nitric Oxide release experiments on Compound **2***

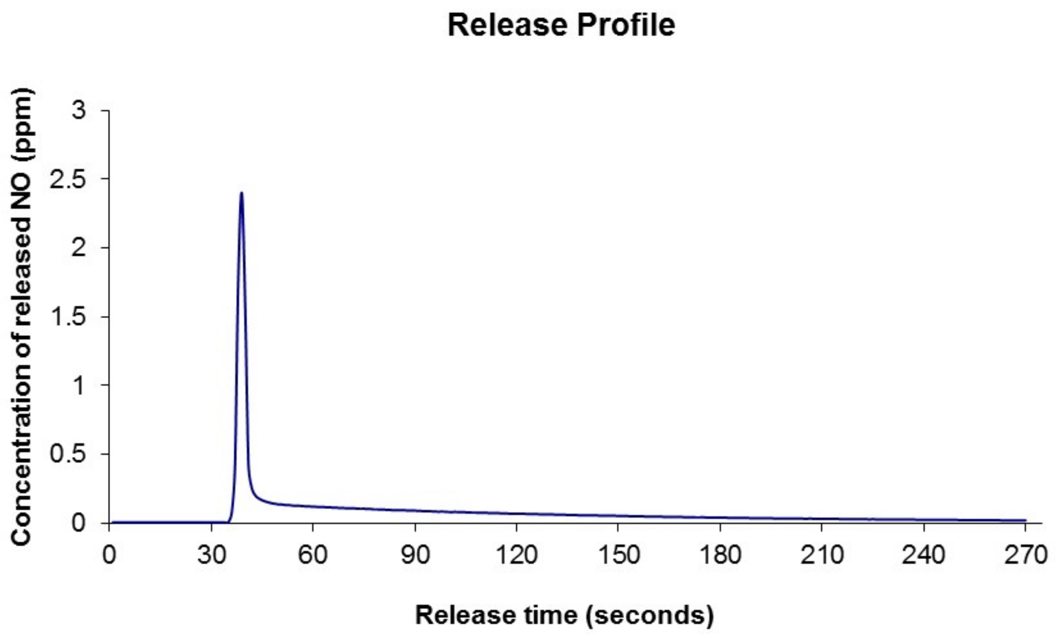

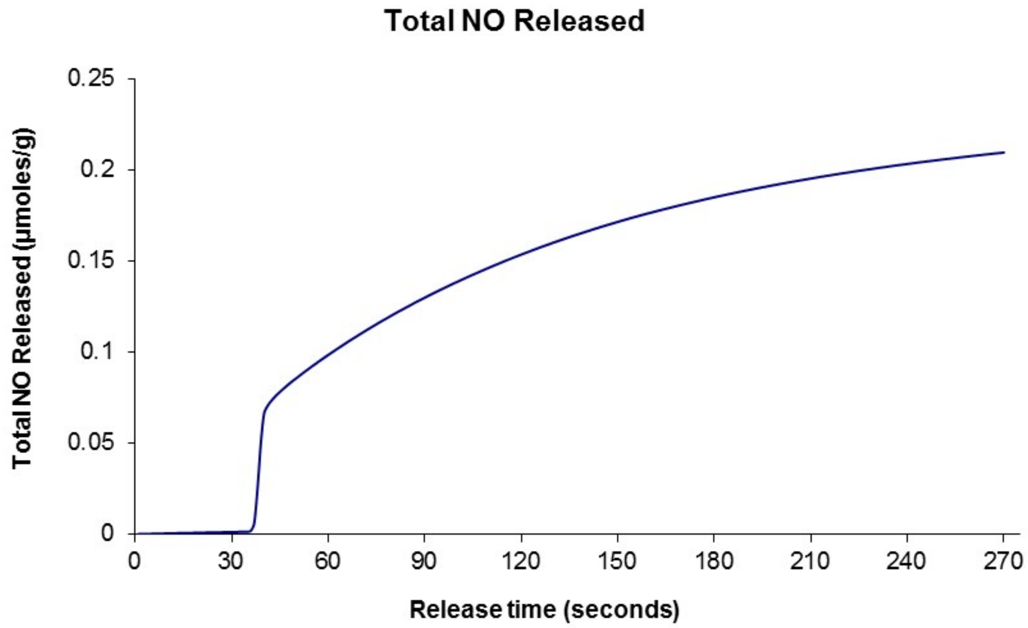

Run 1

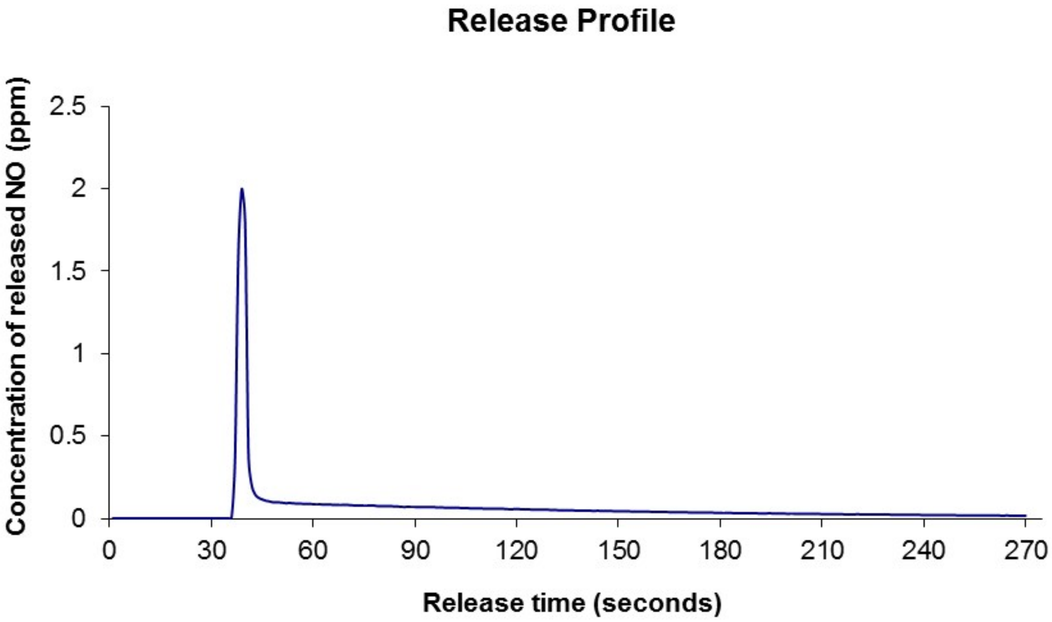

Release Profile
Concentration of released NO (ppm)
2.5
2
1.5
1
0.5
0
0
30
60
90
120
150
180
210
240
270
Release time (seconds)

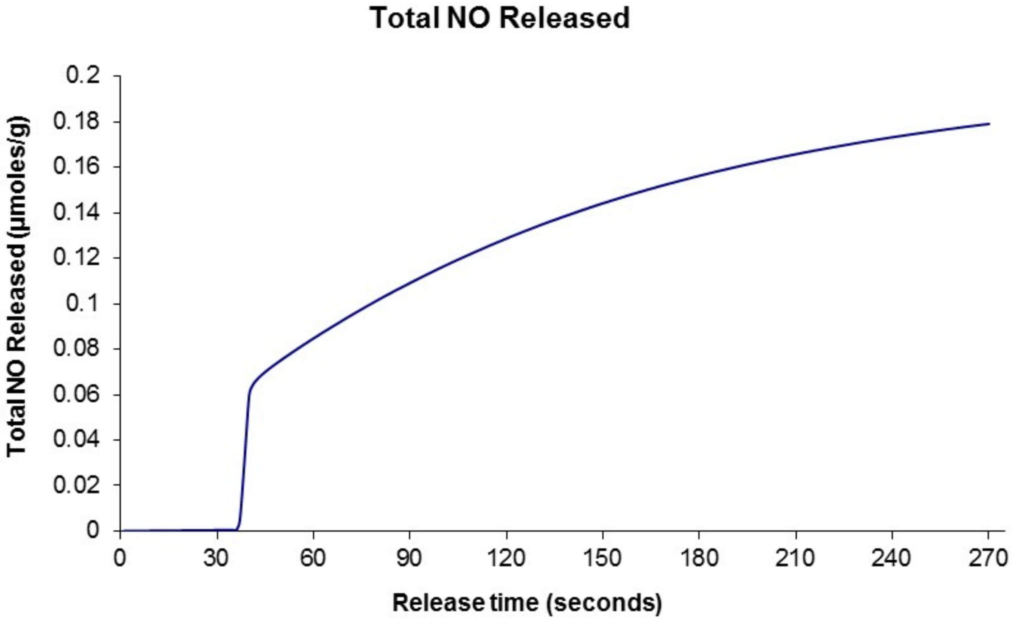

Total NO Released
Total NO Released (µmoles/g)
0.2
0.18
0.16
0.14
0.12
0.1
0.08
0.06
0.04
0.02
0
0
30
60
90
120
150
180
210
240
270
Release time (seconds)

Run 2

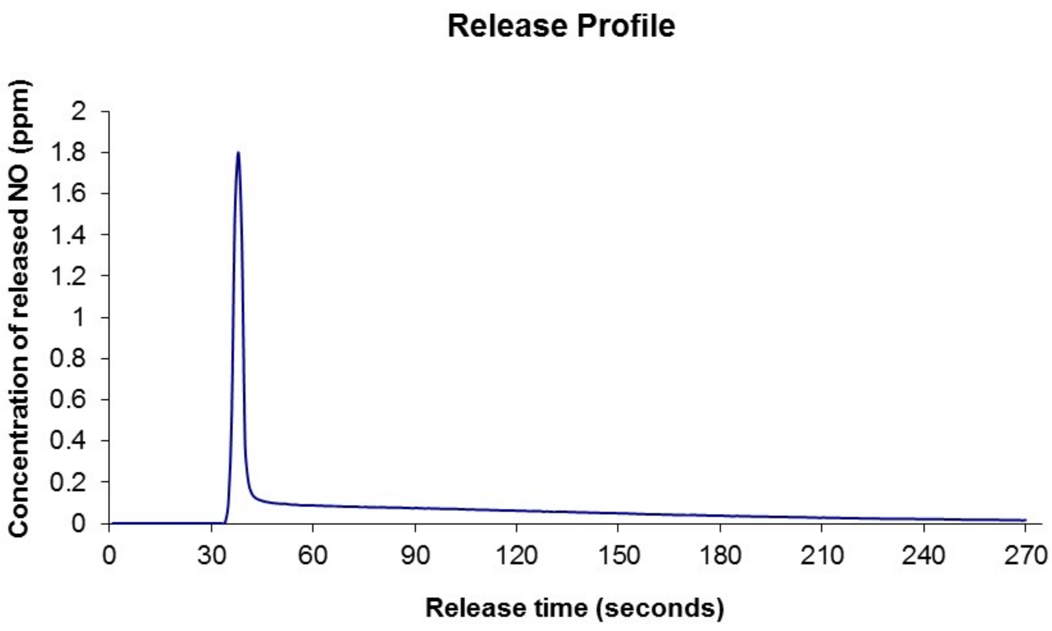

Release Profile
Concentration of released NO (ppm)
2
1.8
1.6
1.4
1.2
1
0.8
0.6
0.4
0.2
0
0
30
60
90
120
150
180
210
240
270
Release time (seconds)

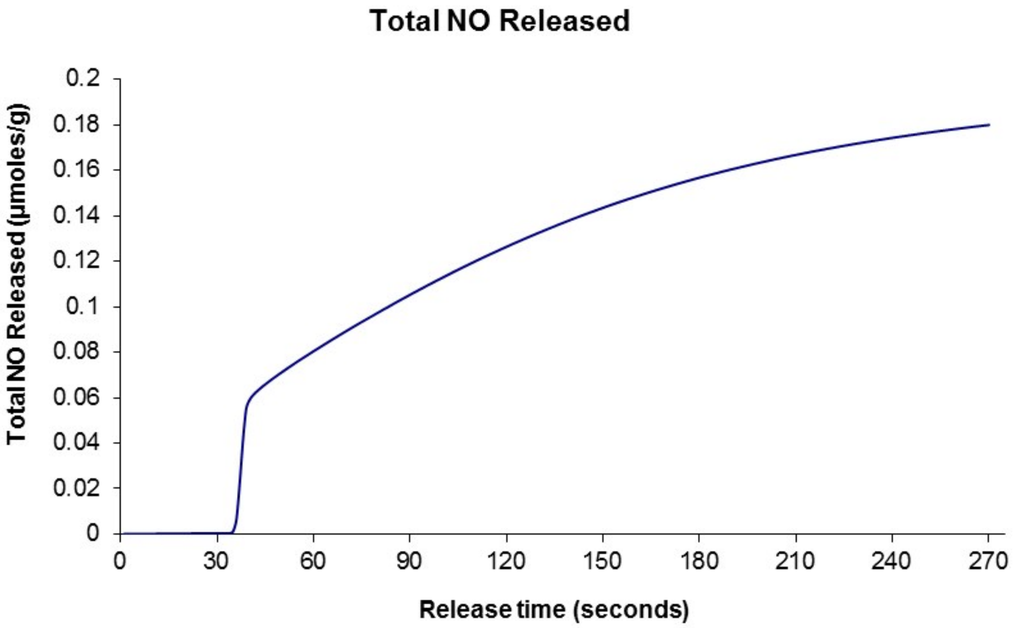

Total NO Released
Total NO Released (µmoles/g)
0.2
0.18
0.16
0.14
0.12
0.1
0.08
0.06
0.04
0.02
0
0
30
60
90
120
150
180
210
240
270
Release time (seconds)

Run 3

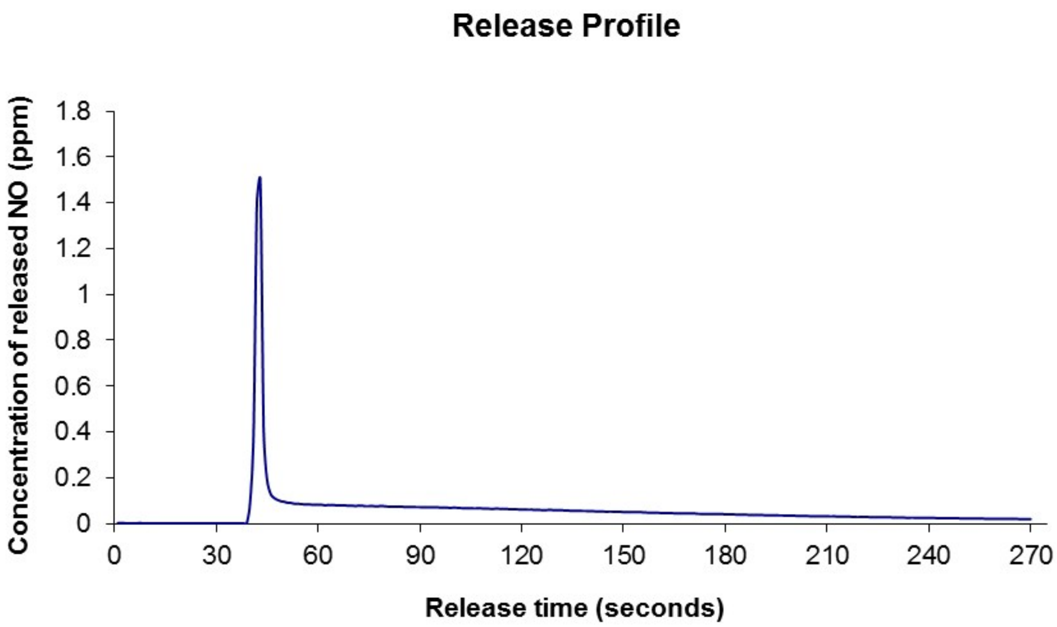
Release Profile
Concentration of released NO (ppm)
1.8
1.6
1.4
1.2
1
0.8
0.6
0.4
0.2
0
0
30
60
90
120
150
180
210
240
270
Release time (seconds)

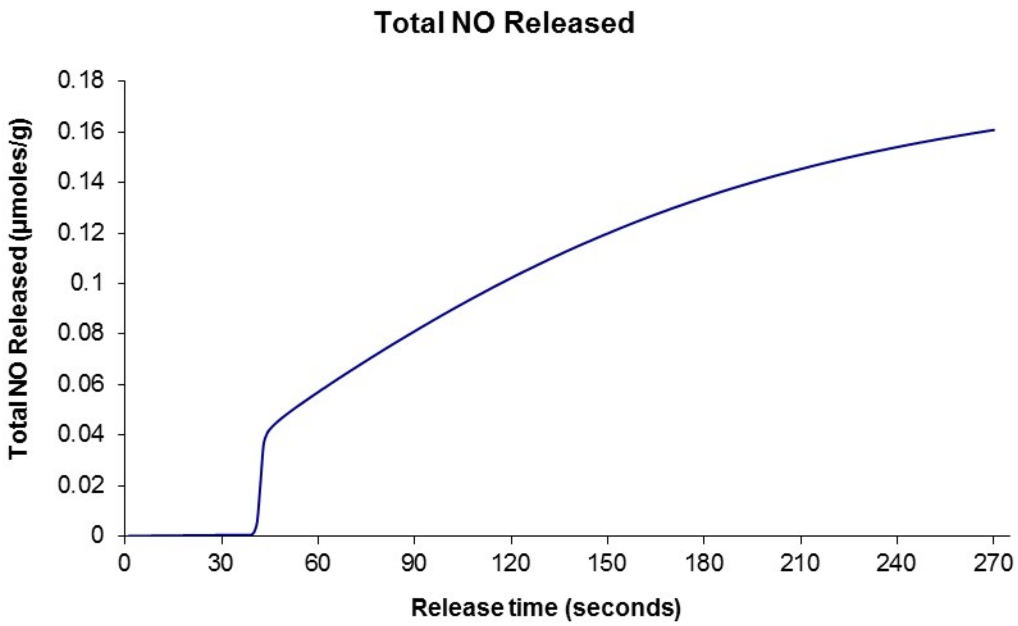
Total NO Released
Total NO Released (µmoles/g)
0.18
0.16
0.14
0.12
0.1
0.08
0.06
0.04
0.02
0
0
30
60
90
120
150
180
210
240
270
Release time (seconds)

Run 4

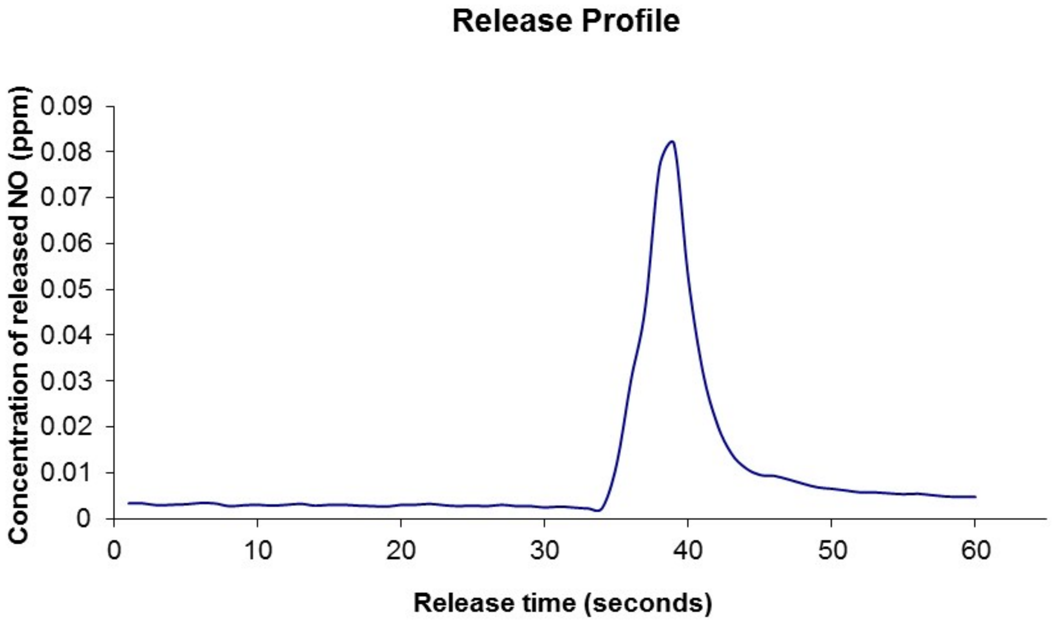

Release Profile
Concentration of released NO (ppm)
0.09
0.08
0.07
0.06
0.05
0.04
0.03
0.02
0.01
0
0
10
20
30
40
50
60
Release time (seconds)

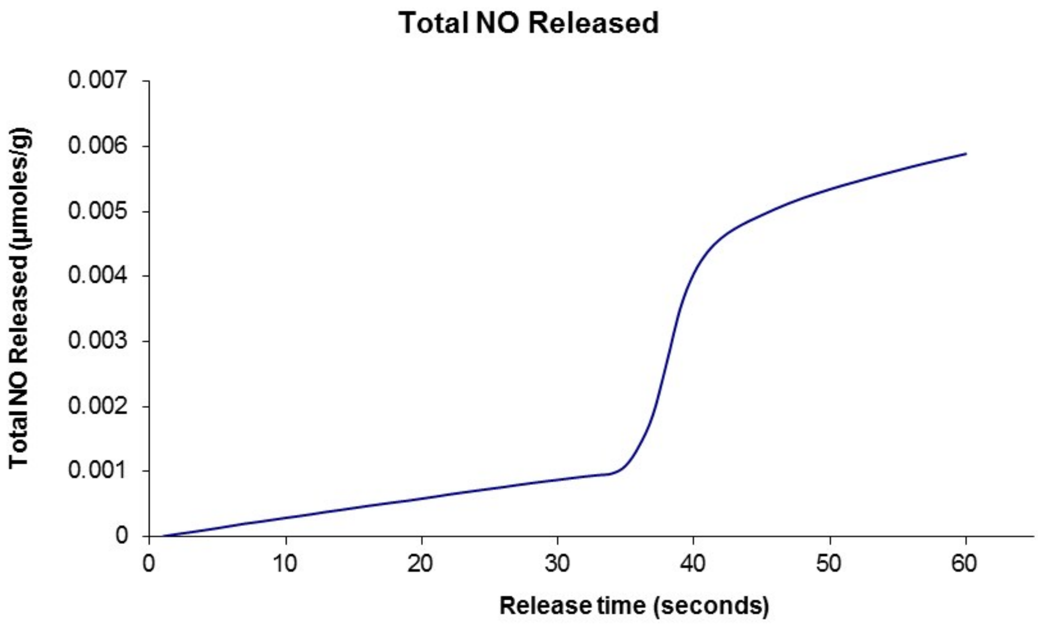

Total NO Released
Total NO Released (µmoles/g)
0.007
0.006
0.005
0.004
0.003
0.002
0.001
0
0
10
20
30
40
50
60
Release time (seconds)

Run 5

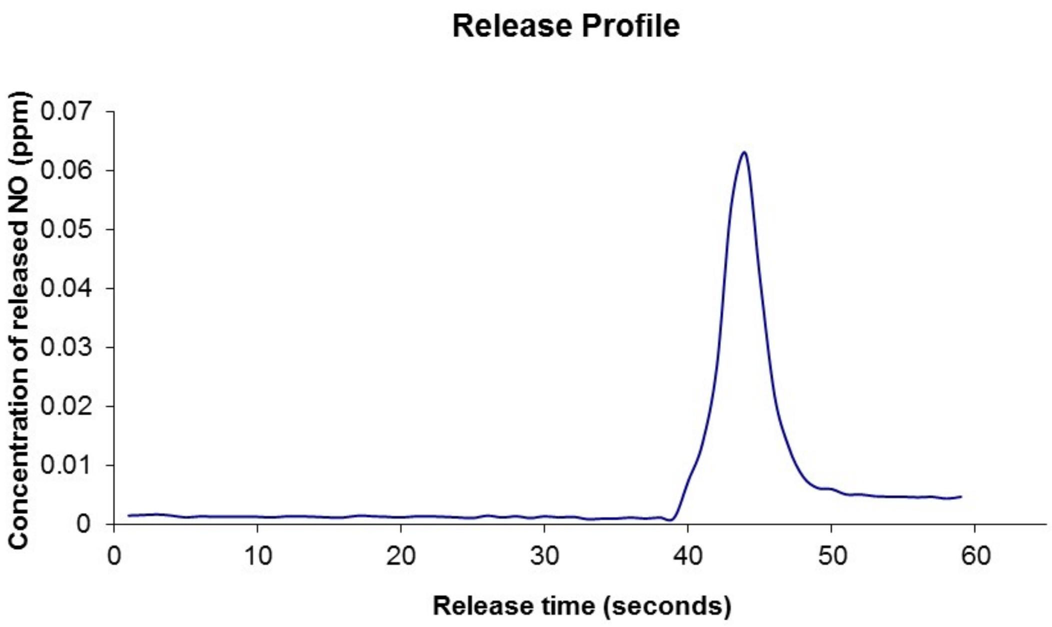
Release Profile
Concentration of released NO (ppm)
0.07
0.06
0.05
0.04
0.03
0.02
0.01
0
0
10
20
30
40
50
60
Release time (seconds)

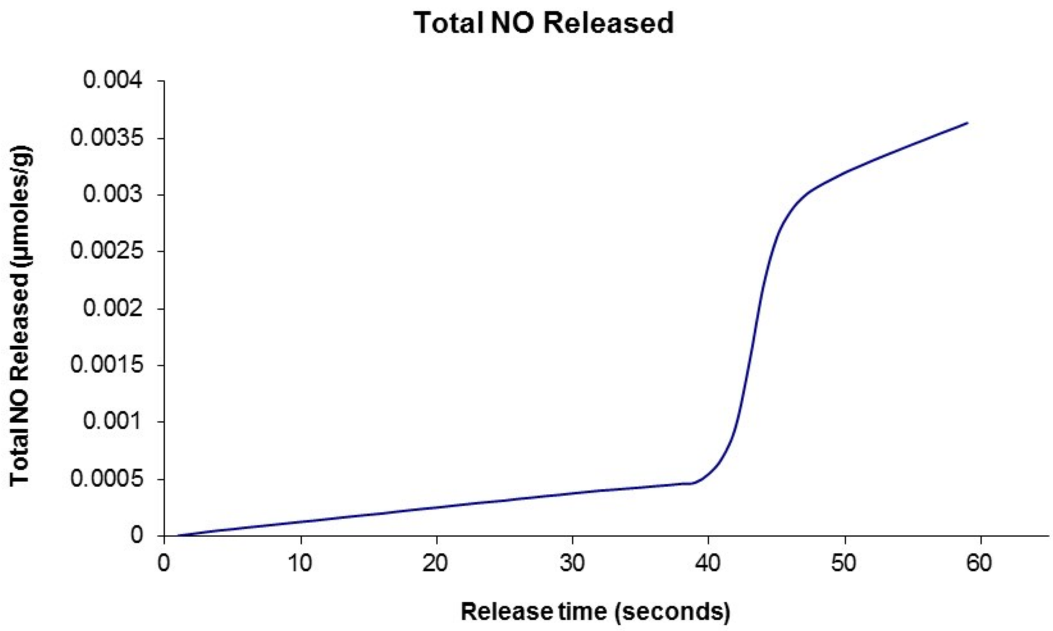
Total NO Released
Total NO Released (µmoles/g)
0.004
0.0035
0.003
0.0025
0.002
0.0015
0.001
0.0005
0
0
10
20
30
40
50
60
Release time (seconds)

Run 6

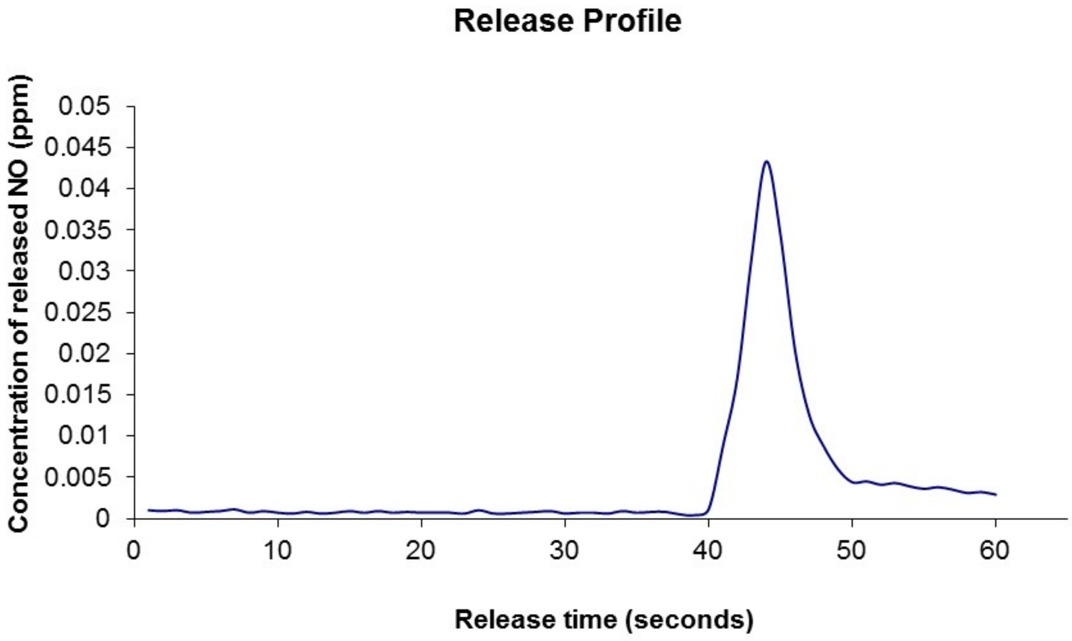
Release Profile
Concentration of released NO (ppm)
0.05
0.045
0.04
0.035
0.03
0.025
0.02
0.015
0.01
0.005
0
0
10
20
30
40
50
60
Release time (seconds)

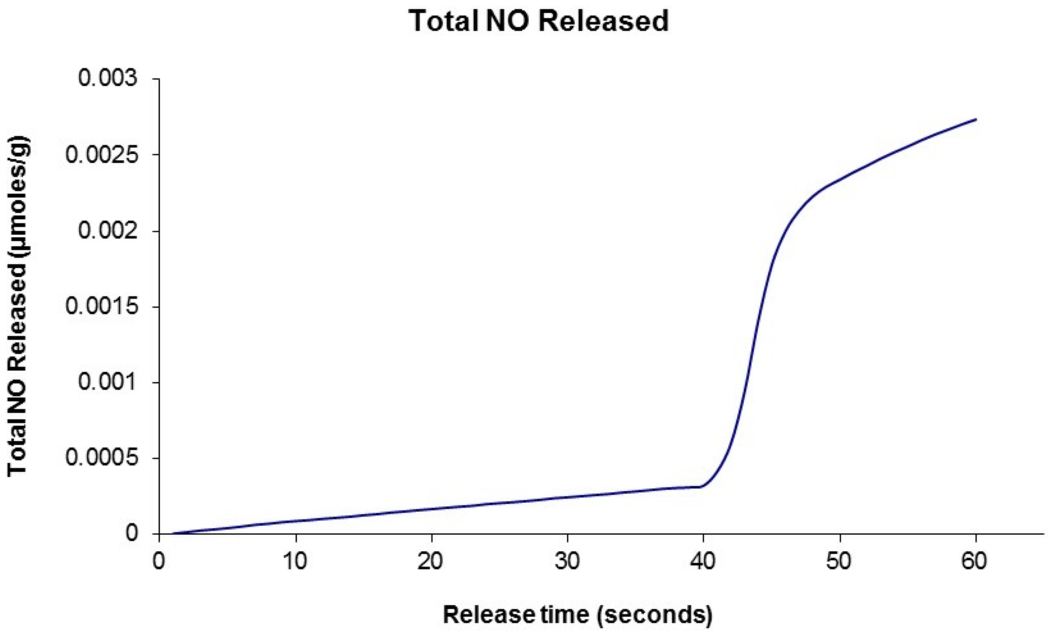
Total NO Released
Total NO Released (µmoles/g)
0.003
0.0025
0.002
0.0015
0.001
0.0005
0
0
10
20
30
40
50
60
Release time (seconds)

Run 7

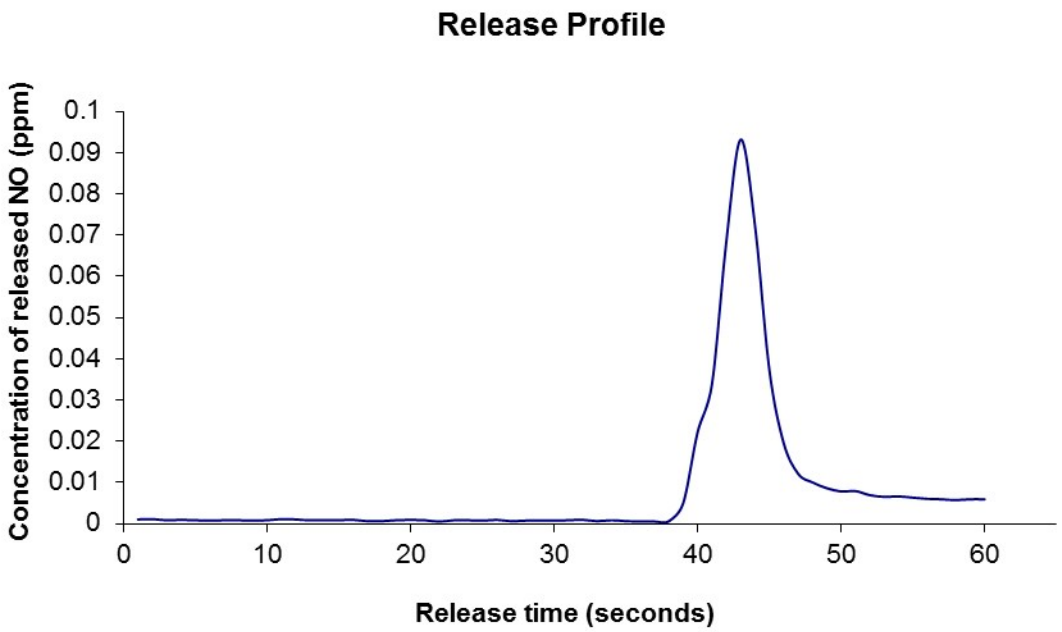
Release Profile
Concentration of released NO (ppm)
0.1
0.09
0.08
0.07
0.06
0.05
0.04
0.03
0.02
0.01
0
0
10
20
30
40
50
60
Release time (seconds)

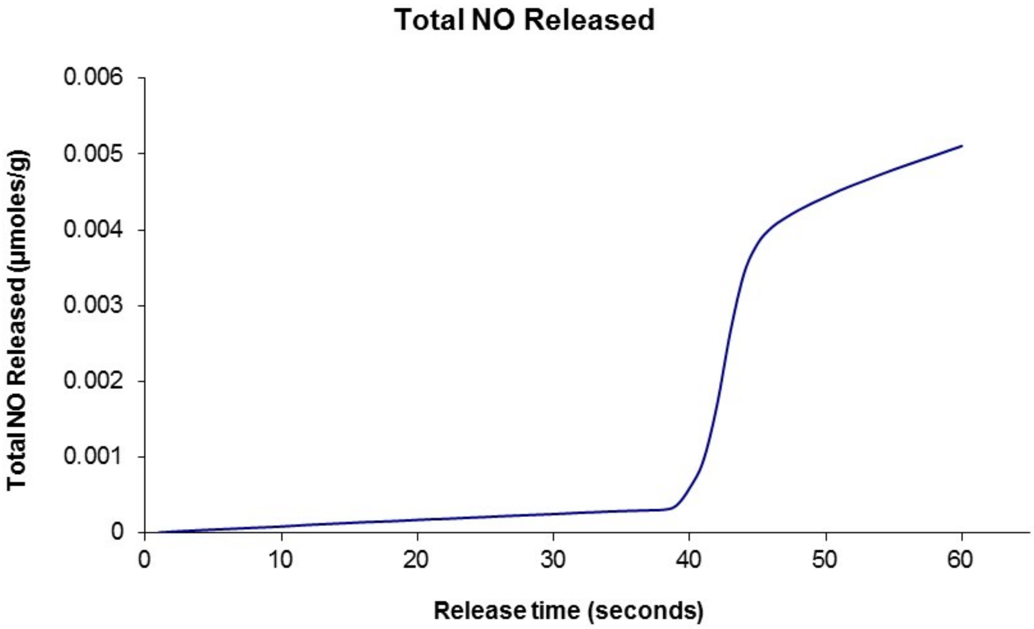
Total NO Released
Total NO Released (µmoles/g)
0.006
0.005
0.004
0.003
0.002
0.001
0
0
10
20
30
40
50
60
Release time (seconds)

Run 8

*S3.2 Plots of Nitric Oxide release experiments on Compound **3***

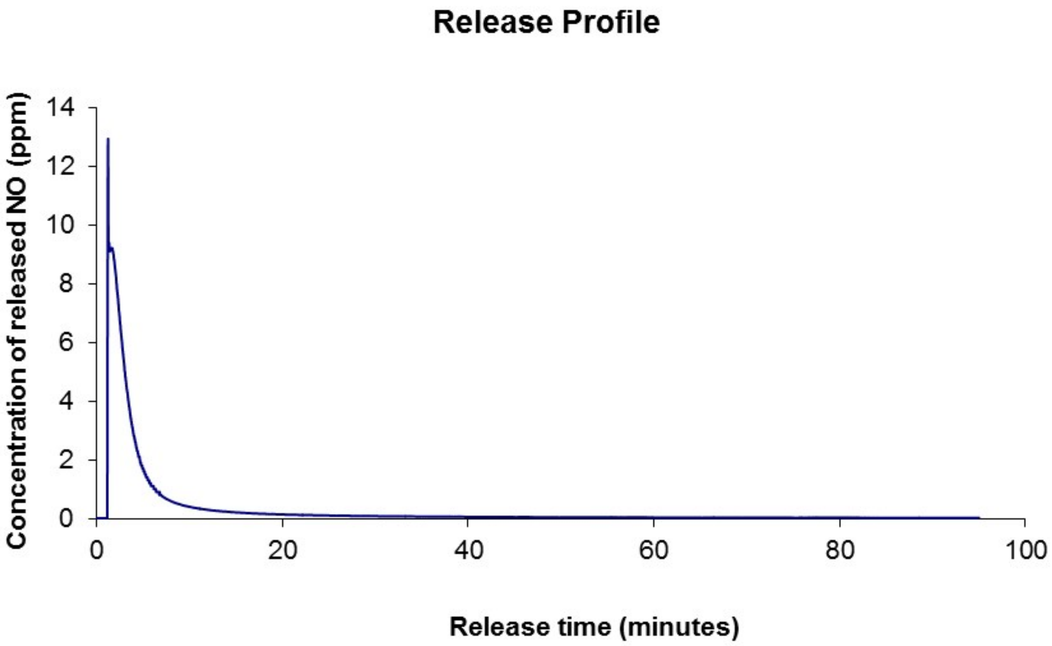

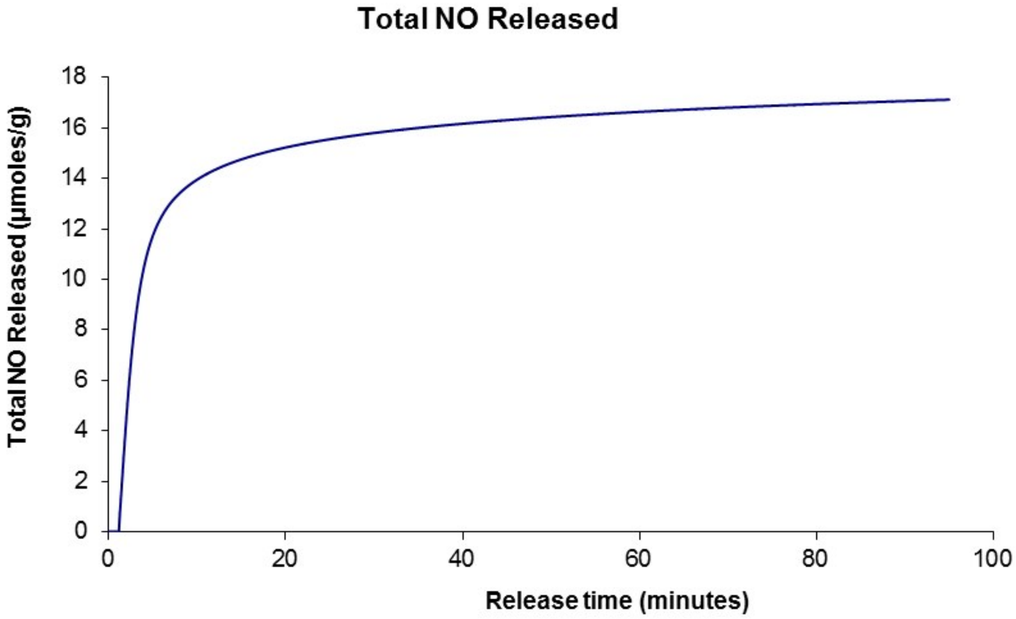

Run 1

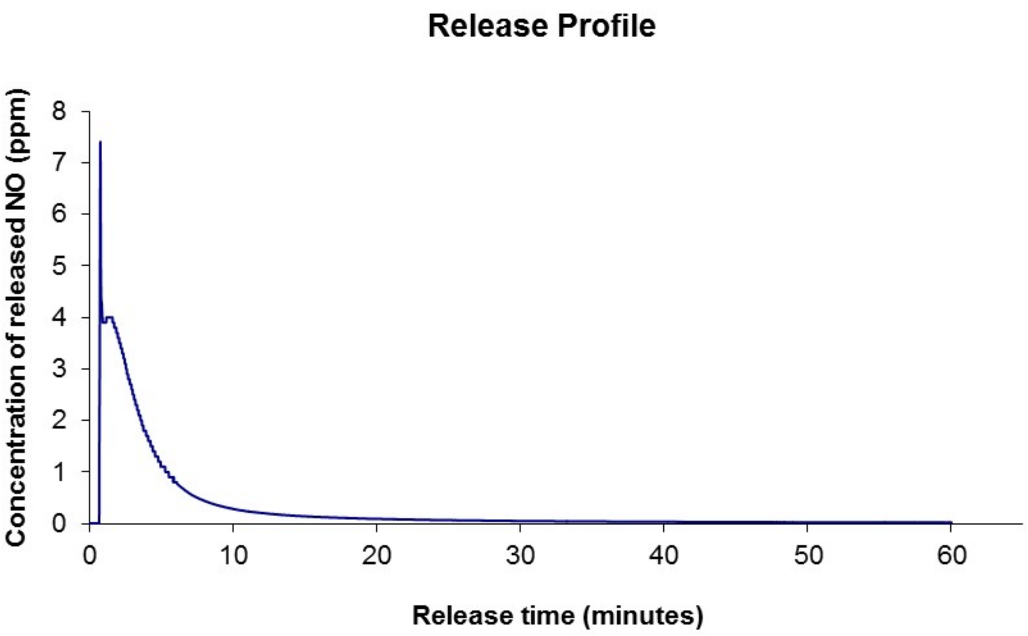
Release Profile
Concentration of released NO (ppm)
0
1
2
3
4
5
6
7
8
0
10
20
30
40
50
60
Release time (minutes)

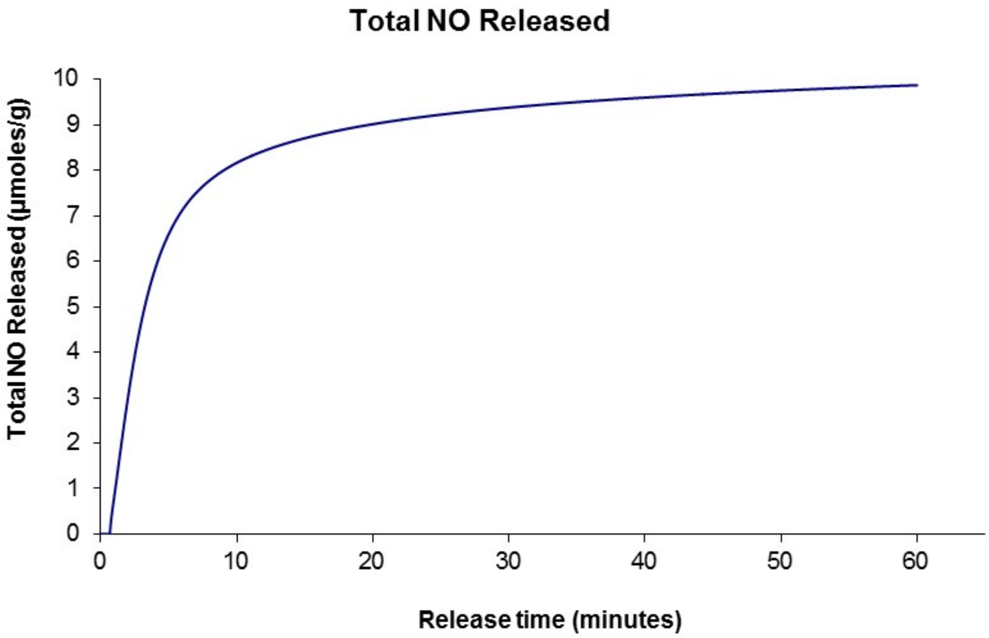
Total NO Released
Total NO Released (µmoles/g)
0
1
2
3
4
5
6
7
8
9
10
0
10
20
30
40
50
60
Release time (minutes)

Run 2

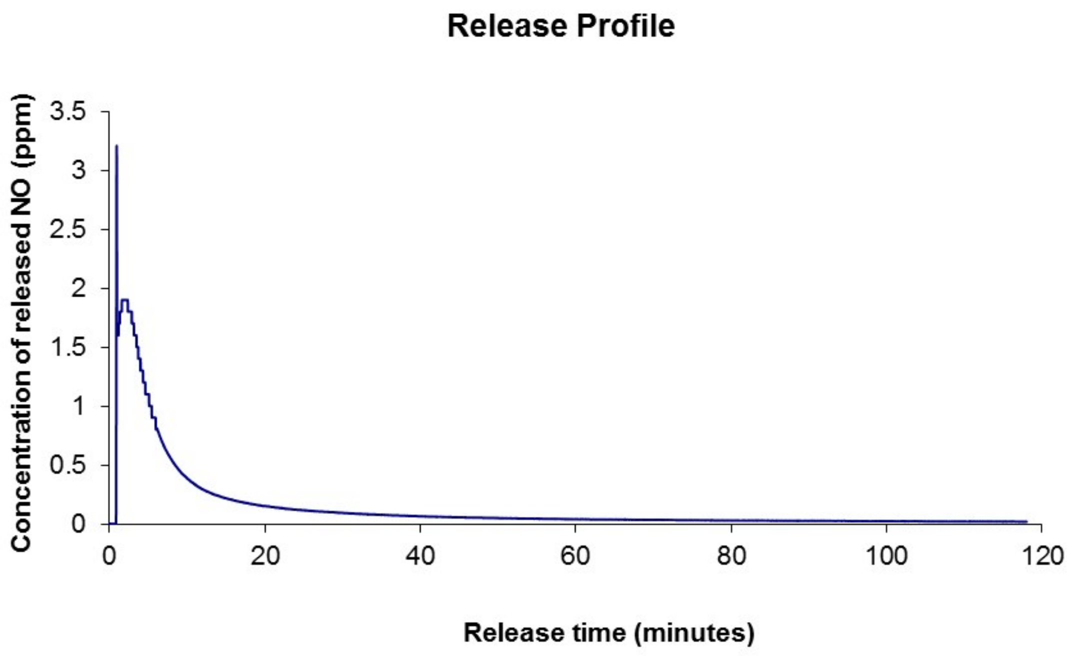
Release Profile
Concentration of released NO (ppm)
3.5
3
2.5
2
1.5
1
0.5
0
0
20
40
60
80
100
120
Release time (minutes)

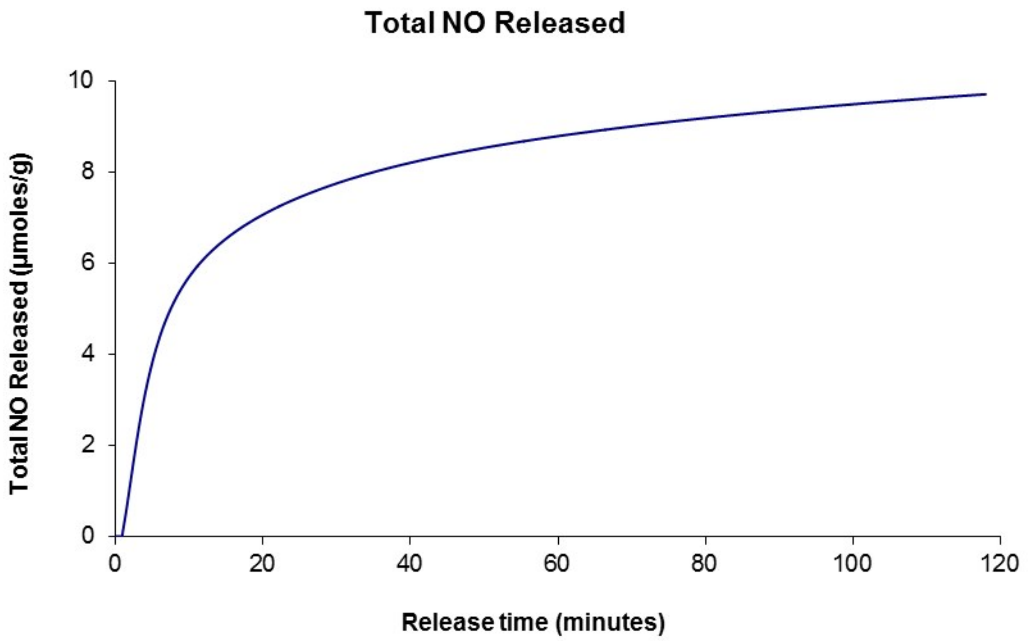
Total NO Released
Total NO Released (µmoles/g)
10
8
6
4
2
0
0
20
40
60
80
100
120
Release time (minutes)

Run 3

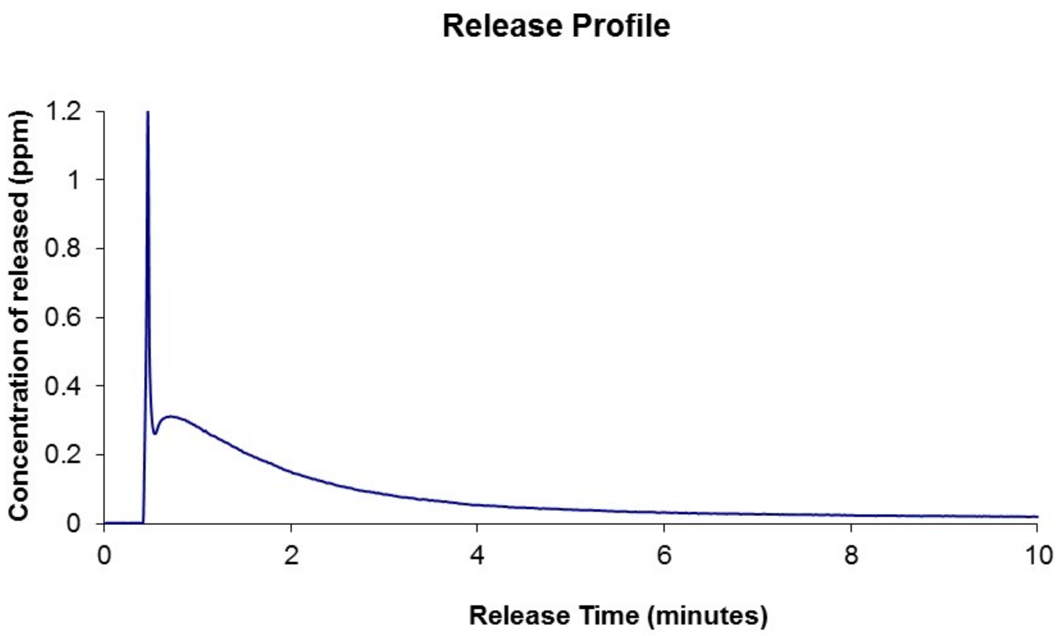
Release Profile
Concentration of released (ppm)
1.2
1
0.8
0.6
0.4
0.2
0
0
2
4
6
8
10
Release Time (minutes)

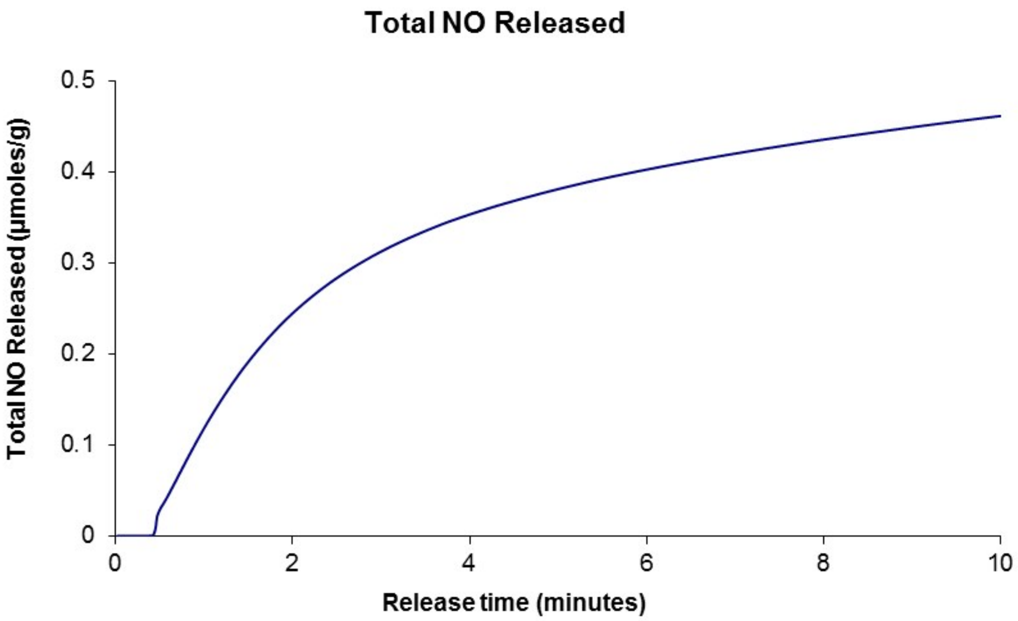
Total NO Released
Total NO Released (µmoles/g)
0.5
0.4
0.3
0.2
0.1
0
0
2
4
6
8
10
Release time (minutes)

Run 4

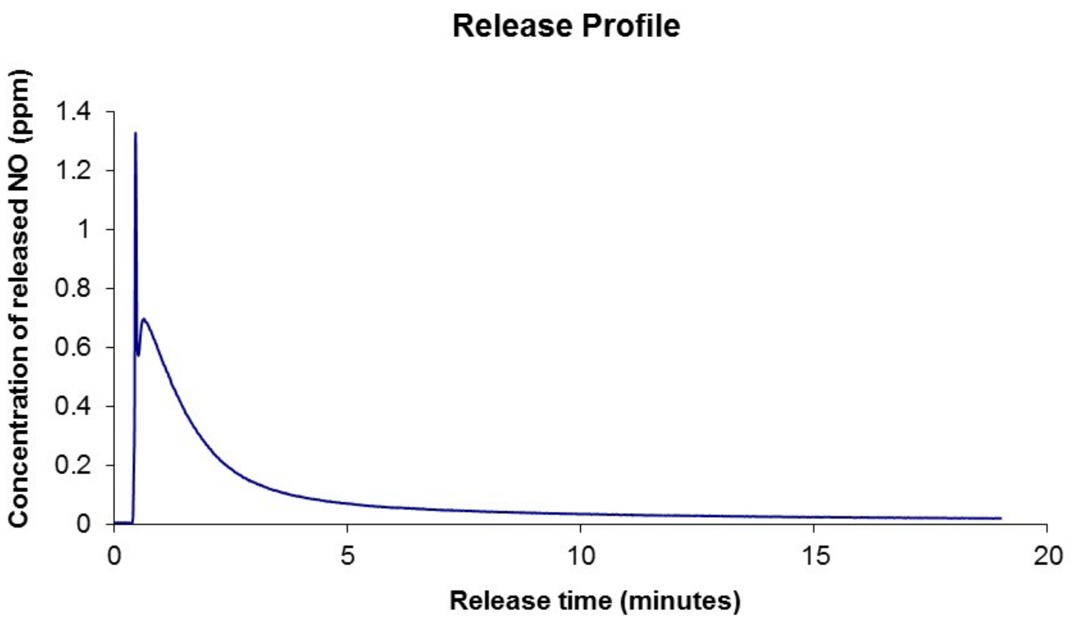
Release Profile
Concentration of released NO (ppm)
1.4
1.2
1
0.8
0.6
0.4
0.2
0
0
5
10
15
20
Release time (minutes)

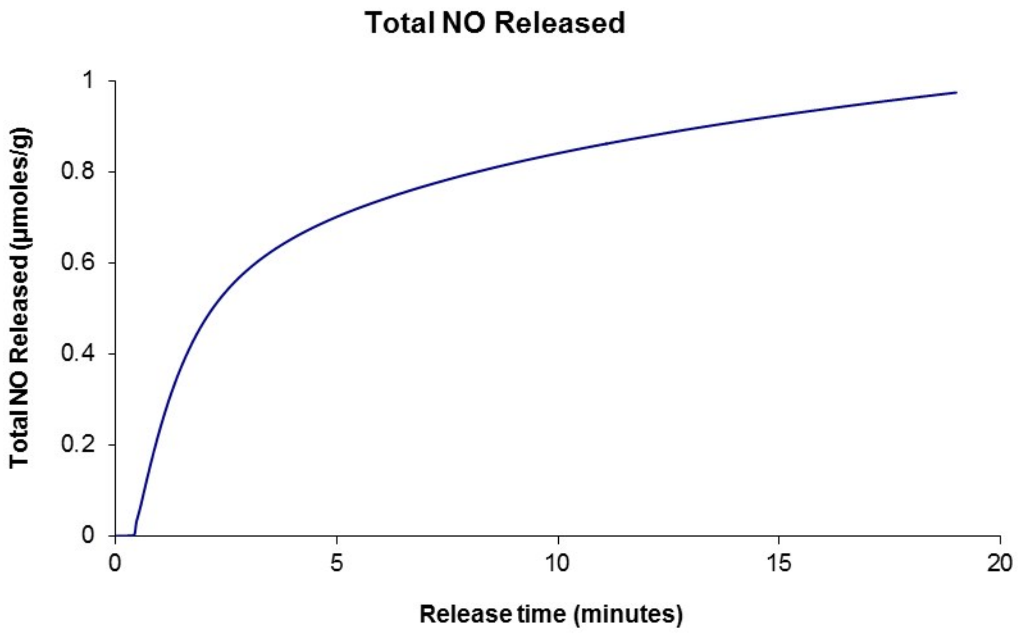
Total NO Released
Total NO Released (µmoles/g)
1
0.8
0.6
0.4
0.2
0
0
5
10
15
20
Release time (minutes)

Run 5

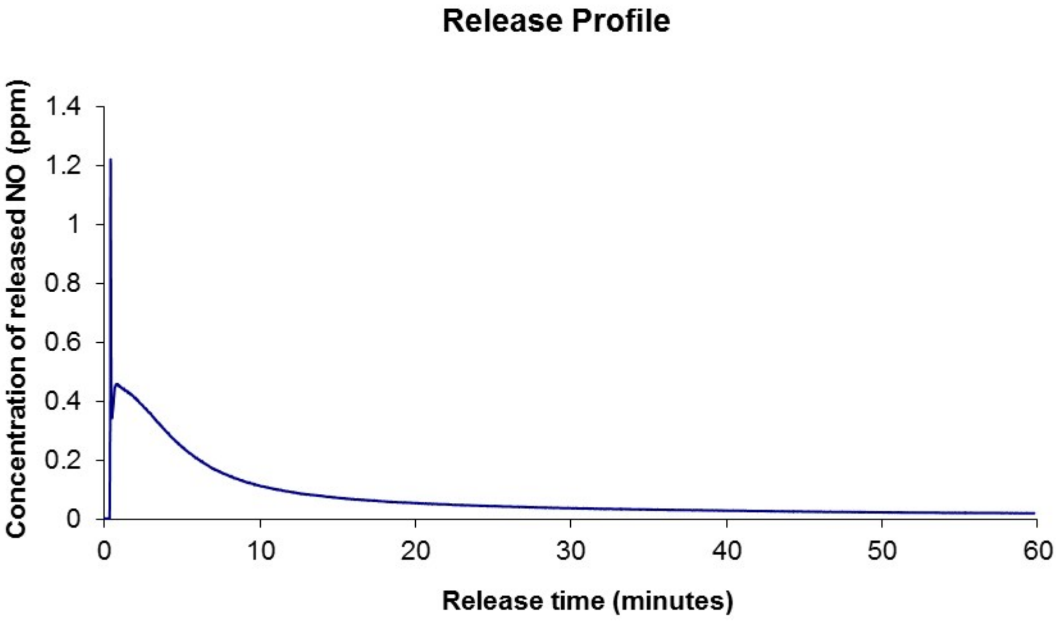
Release Profile
Concentration of released NO (ppm)
1.4
1.2
1
0.8
0.6
0.4
0.2
0
0
10
20
30
40
50
60
Release time (minutes)

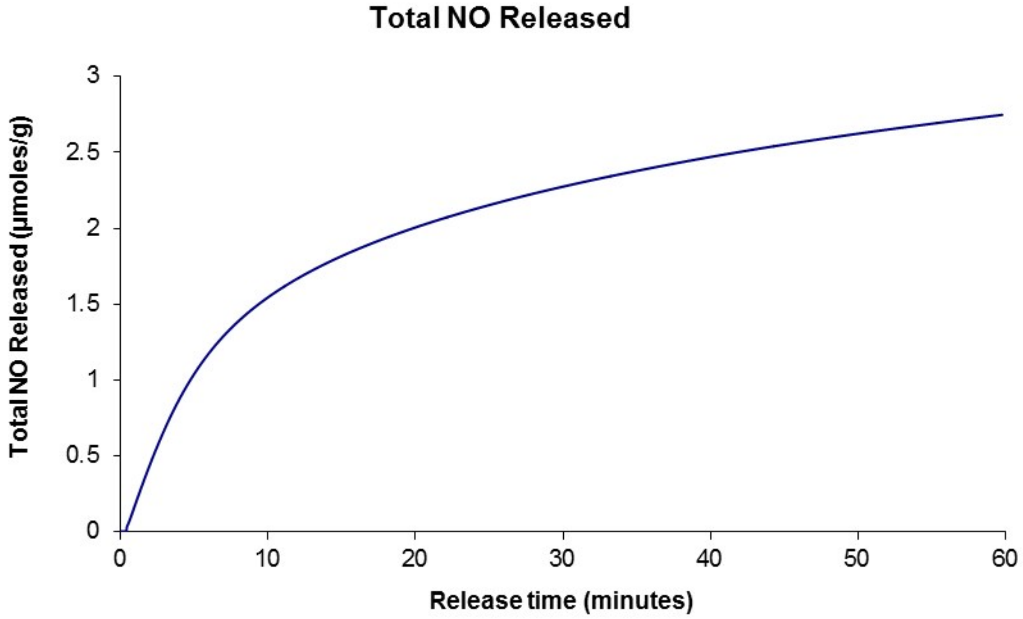
Total NO Released
Total NO Released (µmoles/g)
3
2.5
2
1.5
1
0.5
0
0
10
20
30
40
50
60
Release time (minutes)

Run 6

S4 Comparison of powder diffraction patterns before and after NO-loading and release

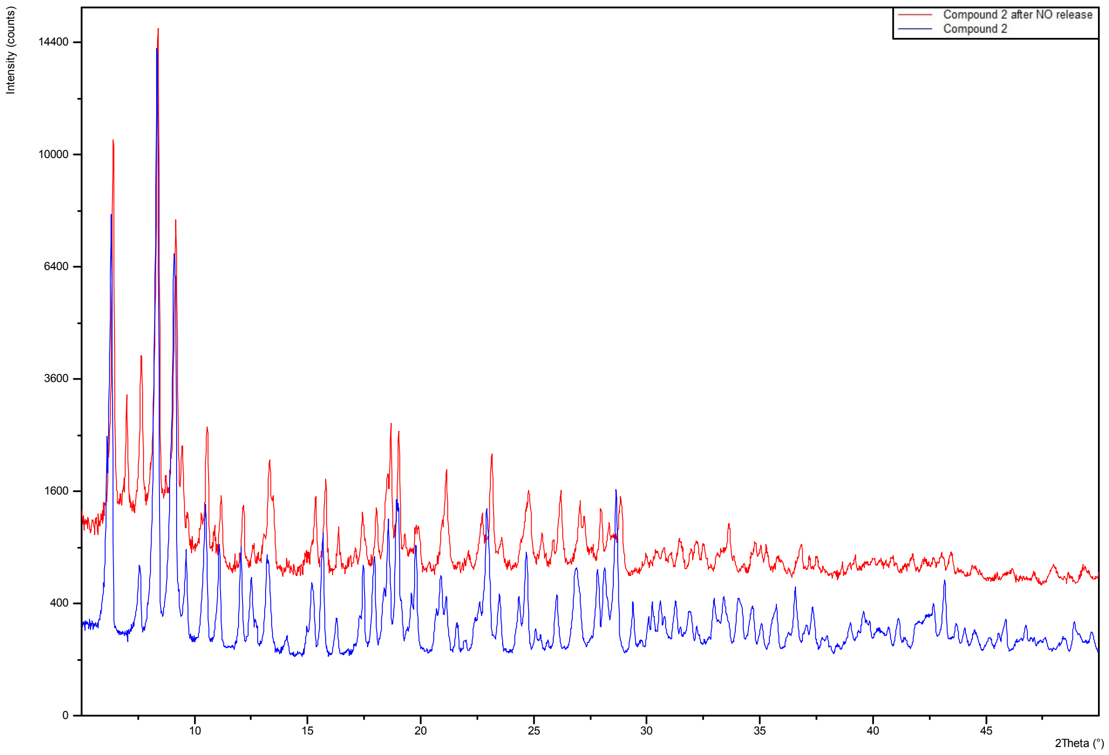

Figure S4.1: Comparison of PXRD patterns of Compound 2 as synthesised (blue) and after (red) dehydration and NO-loading and release experiments. The intensities of the as synthesised powder have been scaled down to 0.3 times their original intensity to for a clear comparison of the patterns.

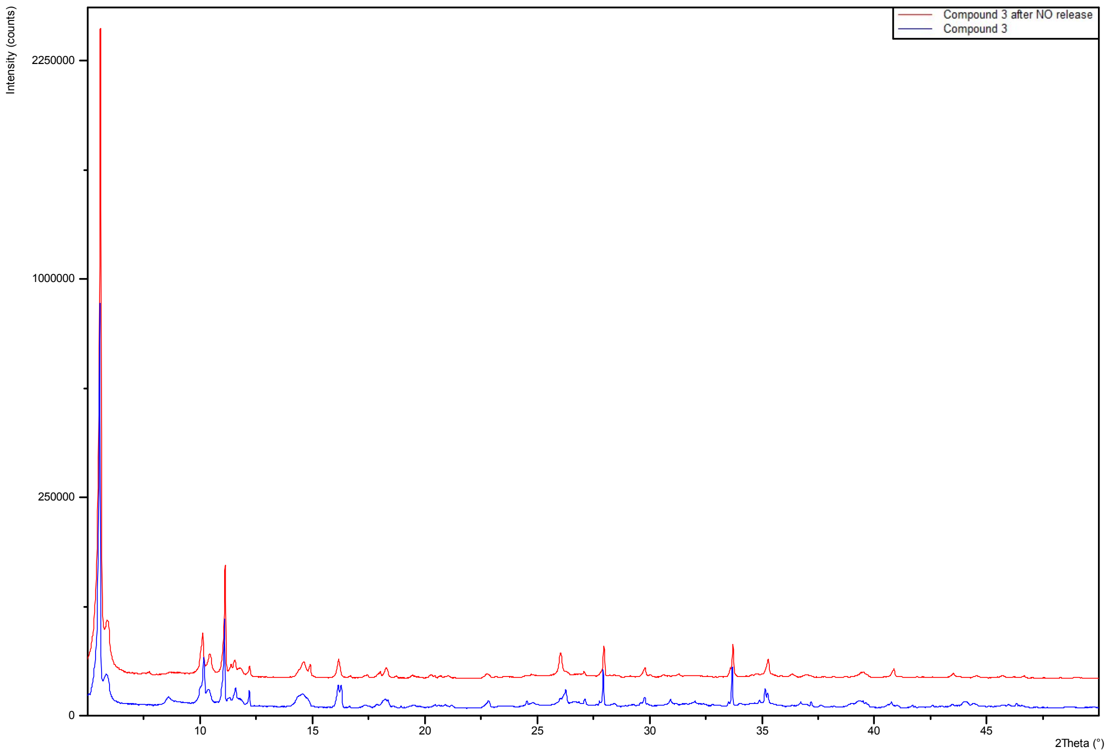

Figure S4.2: Comparison of PXRD patterns of Compound 3 as synthesised (blue) and after (red) dehydration and NO-loading and release experiments. The after NO release trace has been multiplied by a factor of 2.

S5 Selected bond lengths and distances for Compounds 1 to 5

*S5.1 Bond length tables for Compound **1***

Table 1: Selected bond lengths [Å] and angles [°] for Compound 1.

O(1)-Ni(1)	2.043(2)	O(1)-Ni(1)-O(2W)	88.35(11)
O(1W)-Ni(1)	2.078(2)	O(3W)-Ni(1)-O(2W)	94.70(14)
O(3)-Ni(1)^{I}	2.031(2)	O(4W)-Ni(1)-O(2W)	173.30(12)
O(2W)-Ni(1)	2.076(3)	O(3)^{III}-Ni(1)-O(1W)	93.41(10)
O(3W)-Ni(1)	2.047(3)	O(1)-Ni(1)-O(1W)	88.84(10)
O(4W)-Ni(1)	2.051(3)	O(3W)-Ni(1)-O(1W)	177.45(13)
O(6)-Ni(2)	2.019(2)	O(4W)-Ni(1)-O(1W)	86.18(11)
O(5W)-Ni(2)	2.046(3)	O(2W)-Ni(1)-O(1W)	87.61(12)
O(6W)-Ni(2)	2.087(2)	O(6)-Ni(2)-O(8)^{IV}	174.84(14)
O(8)-Ni(2)^{II}	2.021(2)	O(6)-Ni(2)-O(5W)	93.12(12)
O(7W)-Ni(2)	2.117(3)	O(8)^{IV}-Ni(2)-O(5W)	91.97(11)
O(8W)-Ni(2)	2.077(3)	O(6)-Ni(2)-O(8W)	88.57(10)
Ni(1)-O(3)^{III}	2.031(2)	O(8)^{IV}-Ni(2)-O(8W)	90.50(10)
Ni(2)-O(8)^{IV}	2.021(2)	O(5W)-Ni(2)-O(8W)	91.62(12)
		O(6)-Ni(2)-O(6W)	92.80(10)
C(10)-O(6)-Ni(2)	126.6(2)	O(8)^{IV}-Ni(2)-O(6W)	88.14(10)
C(14)-O(8)-Ni(2)^{II}	132.3(2)	O(5W)-Ni(2)-O(6W)	88.34(11)
O(3)^{III}-Ni(1)-O(1)	174.04(12)	O(8W)-Ni(2)-O(6W)	178.64(10)
O(3)^{III}-Ni(1)-O(3W)	87.82(11)	O(6)-Ni(2)-O(7W)	86.17(12)
O(1)-Ni(1)-O(3W)	90.15(11)	O(8)^{IV}-Ni(2)-O(7W)	88.76(12)
O(3)^{III}-Ni(1)-O(4W)	96.64(12)	O(5W)-Ni(2)-O(7W)	178.26(11)
O(1)-Ni(1)-O(4W)	89.01(11)	O(8W)-Ni(2)-O(7W)	89.95(12)
O(3W)-Ni(1)-O(4W)	91.46(14)	O(6W)-Ni(2)-O(7W)	90.11(11)
O(3)^{III}-Ni(1)-O(2W)	86.23(12)		

Symmetry transformations used to generate equivalent atoms:

I: x+1/2, -y+1/2, z **II:** x-1/2, -y+3/2, z **III:** x-1/2, -y+1/2, z

IV: x+1/2, -y+3/2, z

Table 2: Hydrogen bonds for Compound 1 [Å and °].

D-H···A	d(D-H)	d(H···A)	d(D···A)	<(DHA)
O(3W)-H(3A)···O(9W)	0.888(14)	1.959(18)	2.829(5)	166(4)
O(3W)-H(3B)···O(2)	0.889(14)	1.94(3)	2.662(4)	138(3)
O(4W)-H(4B)···O(7)	0.898(14)	1.840(17)	2.727(4)	169(4)
O(5W)-H(5A)···O(4)	0.896(14)	1.93(2)	2.780(4)	157(4)
O(6W)-H(6A)···O(5)	0.884(14)	1.81(2)	2.647(4)	156(4)
O(1W)-H(1B)···O(4)^{III}	0.905(13)	1.800(17)	2.679(4)	164(4)
O(4W)-H(4A)···O(5)^{III}	0.893(14)	2.02(3)	2.784(4)	143(4)
O(1W)-H(1A)···O(8)^{V}	0.881(14)	1.921(15)	2.793(4)	170(4)
O(2W)-H(2A)···O(6)^{V}	0.899(14)	1.96(2)	2.830(4)	162(4)
O(2W)-H(2B)···O(8W)^{V}	0.894(14)	2.19(3)	2.819(4)	127(3)
O(5W)-H(5B)···O(2)^{IV}	0.894(14)	1.874(16)	2.744(4)	164(4)
O(8W)-H(8A)···O(7)^{IV}	0.886(14)	1.88(3)	2.653(4)	145(4)
O(7W)-H(7A)···O(3)^{VI}	0.908(14)	2.09(2)	2.942(4)	156(4)
O(7W)-H(7B)···O(1W)^{VI}	0.887(14)	2.28(3)	3.002(4)	139(4)
O(10W)-H(10B) ···O(6W)	0.916(14)	2.24(4)	2.890(5)	128(4)

Symmetry transformations used to generate equivalent atoms:

I: x+1/2, -y+1/2, z **II:** x-1/2, -y+3/2, z **III:** x-1/2, -y+1/2, z

IV: x+1/2, -y+3/2, z **V:** -x+1/2, y-1/2, z-1/2 **VI:** -x+1, -y+1, z+1/2

*S5.2 Bond length tables for Compound **2***

Table 3: Selected bond lengths [Å] and angles [°] for Compound 2.

O(1)-Zn(1)	2.016(4)
O(1H)-Zn(3)	1.985(4)
O(1H)-Zn(2)^{I}	2.089(5)
O(1H)-Zn(1)^{I}	2.115(5)
O(2A)-Zn(2)	1.973(10)
O(2B)-Zn(2)	1.952(10)
O(1W)-Zn(2)	2.189(6)
O(2H)-Zn(4)	1.974(5)
O(2H)-Zn(5)^{II}	2.077(5)
O(2H)-Zn(6)^{II}	2.126(5)
O(3)-Zn(1)^{I}	2.122(4)
O(3)-Zn(6)^{III}	2.168(4)
O(2WA)-Zn(2)	2.002(11)
O(2WB)-Zn(2)	2.082(19)
O(4)-Zn(3)	1.960(6)
O(3WA)-Zn(5)	2.286(11)
O(3WB)-Zn(5)	2.07(3)
O(5)-Zn(3)	1.953(5)
O(4WA)-Zn(5)	1.871(13)
O(4WB)-Zn(5)	2.10(3)
O(5W)-Zn(5)	2.28(3)
O(7)-Zn(2)^{IV}	1.953(5)
O(8)-Zn(1)^{IV}	2.025(5)
O(9)-Zn(6)^{III}	2.086(5)
O(10)-Zn(3)	1.957(5)
O(11)-Zn(1)^{V}	2.074(5)
O(12)-Zn(4)	1.956(5)
O(13)-Zn(6)^{II}	2.137(4)
O(13)-Zn(1)^{V}	2.201(4)
O(14)-Zn(4)	1.954(6)
O(15)-Zn(6)^{VI}	2.037(5)
O(16A)-Zn(5)^{VI}	1.968(8)
O(16B)-Zn(5)^{VI}	1.945(16)
O(17)-Zn(4)	1.960(5)
O(19)-Zn(5)	1.969(6)
O(20)-Zn(6)	2.006(5)
Zn(3)-O(1H)-Zn(2)^{I}	118.4(2)
Zn(3)-O(1H)-Zn(1)^{I}	113.7(2)
Zn(2)^{I}-O(1H)-Zn(1)^{I}	103.26(19)

Zn(4)-O(2H)-Zn(5)^{II}	119.0(2)
Zn(4)-O(2H)-Zn(6)^{II}	113.3(2)
Zn(5)^{II}-O(2H)-Zn(6)^{II}	102.4(2)
Zn(1)^{I}-O(3)-Zn(6)^{III}	103.22(17)
Zn(6)^{II}-O(13)-Zn(1)^{V}	101.62(17)
O(1)-Zn(1)-O(8)^{VII}	105.8(2)
O(1)-Zn(1)-O(11)^{VIII}	89.81(19)
O(8)^{VII}-Zn(1)-O(11)^{VIII}	92.3(2)
O(1)-Zn(1)-O(1H)^{IX}	96.15(19)
O(8)^{VII}-Zn(1)-O(1H)^{IX}	89.91(19)
O(11)^{VIII}-Zn(1)-O(1H)^{IX}	172.80(18)
O(1)-Zn(1)-O(3)^{IX}	163.20(19)
O(8)^{VII}-Zn(1)-O(3)^{IX}	89.21(19)
O(11)^{VIII}-Zn(1)-O(3)^{IX}	81.86(19)
O(1H)^{IX}-Zn(1)-O(3)^{IX}	91.35(18)
O(1)-Zn(1)-O(13)^{VIII}	87.76(18)
O(8)^{VII}-Zn(1)-O(13)^{VIII}	166.48(18)
O(11)^{VIII}-Zn(1)-O(13)^{VIII}	87.5(2)
O(1H)^{IX}-Zn(1)-O(13)^{VIII}	88.72(18)
O(3)^{IX}-Zn(1)-O(13)^{VIII}	77.38(16)
O(2B)-Zn(2)-O(7)#7	132.6(8)
O(7)^{VII}-Zn(2)-O(2A)	121.8(7)
O(2B)-Zn(2)-O(2WA)	120.6(8)
O(7)^{VII}-Zn(2)-O(2WA)	106.0(5)
O(2A)-Zn(2)-O(2WA)	130.0(7)
O(2B)-Zn(2)-O(2WB)	98.4(10)
O(7)^{VII}-Zn(2)-O(2WB)	128.7(7)
O(2A)-Zn(2)-O(2WB)	108.6(9)
O(2B)-Zn(2)-O(1H)^{IX}	92.9(5)
O(7)^{VII}-Zn(2)-O(1H)^{IX}	92.4(2)
O(2A)-Zn(2)-O(1H)^{IX}	97.9(4)
O(2WA)-Zn(2)-O(1H)^{IX}	93.7(4)
O(2WB)-Zn(2)-O(1H)^{IX}	89.6(5)
O(2B)-Zn(2)-O(1W)	87.8(5)
O(7)^{VII}-Zn(2)-O(1W)	86.3(2)
O(2A)-Zn(2)-O(1W)	82.6(4)
O(2WA)-Zn(2)-O(1W)	87.0(4)
O(2WB)-Zn(2)-O(1W)	91.4(5)
O(1H)^{IX}-Zn(2)-O(1W)	178.7(2)
O(5)-Zn(3)-O(10)	106.9(2)

O(5)-Zn(3)-O(4)	110.2(2)
O(10)-Zn(3)-O(4)	110.0(2)
O(5)-Zn(3)-O(1H)	108.2(2)
O(10)-Zn(3)-O(1H)	113.1(2)
O(4)-Zn(3)-O(1H)	108.5(2)
O(14)-Zn(4)-O(12)	110.5(2)
O(14)-Zn(4)-O(17)	108.3(3)
O(12)-Zn(4)-O(17)	109.6(2)
O(14)-Zn(4)-O(2H)	108.8(2)
O(12)-Zn(4)-O(2H)	114.2(2)
O(17)-Zn(4)-O(2H)	105.1(2)
O(4WA)-Zn(5)-O(16A)^{X}	121.8(5)
O(4WA)-Zn(5)-O(19)	109.2(5)
O(16B)^{X}-Zn(5)-O(19)	108.5(6)
O(16A)^{X}-Zn(5)-O(19)	126.4(3)
O(16B)^{X}-Zn(5)-O(3WB)	82.8(11)
O(19)-Zn(5)-O(3WB)	85.5(9)
O(4WA)-Zn(5)-O(2H)^{II}	95.8(4)
O(16B)^{X}-Zn(5)-O(2H)^{II}	109.0(5)
O(16A)^{X}-Zn(5)-O(2H)^{II}	95.5(3)
O(19)-Zn(5)-O(2H)^{II}	94.7(2)
O(3WB)-Zn(5)-O(2H)^{II}	167.3(10)
O(16B)^{X}-Zn(5)-O(4WB)	122.3(10)
O(19)-Zn(5)-O(4WB)	88.3(8)
O(2H)^{II}-Zn(5)-O(4WB)	124.5(8)
O(16B)^{X}-Zn(5)-O(5W)	82.5(9)
O(19)-Zn(5)-O(5W)	168.6(7)
O(3WB)-Zn(5)-O(5W)	93.0(11)
O(2H)^{II}-Zn(5)-O(5W)	84.2(7)
O(4WB)-Zn(5)-O(5W)	83.0(11)
O(4WA)-Zn(5)-O(3WA)	91.1(5)
O(16A)^{X}-Zn(5)-O(3WA)	79.9(4)
O(19)-Zn(5)-O(3WA)	84.0(3)
O(2H)^{II}-Zn(5)-O(3WA)	173.0(3)
O(20)-Zn(6)-O(15)^{X}	104.7(2)
O(20)-Zn(6)-O(9)^{XI}	91.5(2)
O(15)^{X}-Zn(6)-O(9)^{XI}	90.1(2)
O(20)-Zn(6)-O(2H)^{II}	91.04(19)
O(15)^{X}-Zn(6)-O(2H)^{II}	95.5(2)
O(9)^{XI}-Zn(6)-O(2H)^{II}	173.06(18)
O(20)-Zn(6)-O(13)^{II}	88.7(2)
O(15)^{X}-Zn(6)-O(13)^{II}	164.4(2)
O(9)^{XI}-Zn(6)-O(13)^{II}	81.64(19)
O(2H)^{II}-Zn(6)-O(13)^{II}	91.96(19)
O(20)-Zn(6)-O(3)^{XI}	166.53(19)
O(15)^{X}-Zn(6)-O(3)^{XI}	88.56(18)
O(9)^{XI}-Zn(6)-O(3)^{XI}	86.21(19)
O(2H)^{II}-Zn(6)-O(3)^{XI}	89.82(18)
O(13)^{II}-Zn(6)-O(3)^{XI}	77.79(16)

Symmetry transformations used to generate equivalent atoms:

I: x, y, z-1 **II:** -x, -y+1, z **III:** -x+1/2, y-1/2, z-1/2

IV: -x+1, -y+1, z-1 **V:** x-1/2, -y+1/2, z-1/2 **VI:** -x, -y+1, z-1

VII: -x+1, -y+1, z+1 **VIII:** x+1/2, -y+1/2, z+1/2 **IX:** x, y, z+1

X: -x, -y+1, z+1 **XI:** -x+1/2, y+1/2, z+1/2

*S5.3 Bond length tables for Compound **3***

Table 4: Selected bond lengths [Å] and angles [°] for Compound **3**.

O(1)-Zn(2)	2.021(8)	O(9)-Zn(1)-Zn(2)	150.3(2)
O(1H)-Zn(1)	1.990(7)	O(7)-Zn(1)-Zn(2)	88.4(2)
O(1H)-Zn(3)	2.023(7)	O(5)-Zn(1)-Zn(2)	84.3(2)
O(1H)-Zn(2)	2.087(7)	O(1)-Zn(2)-O(6)	163.9(3)
O(2)-Zn(3)^{III}	1.941(8)	O(1)-Zn(2)-O(8)	95.0(3)
O(1W)-Zn(2)	2.259(8)	O(6)-Zn(2)-O(8)	100.3(3)
O(3)-Zn(3)^{III}	2.113(7)	O(1)-Zn(2)-O(1H)	93.0(3)
O(4)-Zn(3)^{III}	2.244(8)	O(6)-Zn(2)-O(1H)	91.8(3)
O(4)-Zn(2)	2.283(7)	O(8)-Zn(2)-O(1H)	89.9(3)
O(5)-Zn(1)	1.976(7)	O(1)-Zn(2)-O(1W)	85.1(3)
O(6)-Zn(2)	2.025(8)	O(6)-Zn(2)-O(1W)	79.0(3)
O(7)-Zn(1)	1.960(8)	O(8)-Zn(2)-O(1W)	171.9(3)
O(8)-Zn(2)	2.076(8)	O(1H)-Zn(2)-O(1W)	98.2(3)
O(9)-Zn(1)	1.952(8)	O(1)-Zn(2)-O(4)	92.0(3)
O(10)-Zn(3)	2.021(8)	O(6)-Zn(2)-O(4)	84.3(3)
Zn(1)-Zn(2)	3.1449(17)	O(8)-Zn(2)-O(4)	86.2(3)
Zn(3)-O(2)^{VI}	1.941(8)	O(1H)-Zn(2)-O(4)	173.9(3)
Zn(3)-O(3)^{VI}	2.113(7)	O(1W)-Zn(2)-O(4)	85.8(3)
Zn(3)-O(4)^{VI}	2.244(8)	O(2)^{VI}-Zn(3)-O(10)	98.2(4)
		O(2)^{VI}-Zn(3)-O(1H)	99.3(3)
Zn(1)-O(1H)-Zn(3)	106.3(3)	O(10)-Zn(3)-O(1H)	98.0(3)
Zn(1)-O(1H)-Zn(2)	100.9(3)	O(2)^{VI}-Zn(3)-O(3)^{VI}	156.3(3)
Zn(3)-O(1H)-Zn(2)	127.3(4)	O(10)-Zn(3)-O(3)^{VI}	97.6(3)
Zn(3)^{III}-O(4)-Zn(2)	98.9(3)	O(1H)-Zn(3)-O(3)^{VI}	95.8(3)
O(9)-Zn(1)-O(7)	117.6(3)	O(2)^{VI}-Zn(3)-O(4)^{VI}	98.2(3)
O(9)-Zn(1)-O(5)	102.7(4)	O(10)-Zn(3)-O(4)^{VI}	150.5(3)
O(7)-Zn(1)-O(5)	101.9(4)	O(1H)-Zn(3)-O(4)^{VI}	103.3(3)
O(9)-Zn(1)-O(1H)	111.9(3)	O(3)^{VI}-Zn(3)-O(4)^{VI}	60.4(3)
O(7)-Zn(1)-O(1H)	110.3(3)	O(2)^{VI}-Zn(3)-C(13)^{VI}	128.2(3)
O(5)-Zn(1)-O(1H)	111.7(3)		

Symmetry transformations used to generate equivalent atoms:

I: -x, -y+2, z **II:** -x+1, -y+2, z **III:** -x+1/2, y+1/2, -z

IV: x, y+1, z **V:** x, y-1, z **VI:** -x+1/2, y-1/2, -z

Table 5: Hydrogen bonds for Compound **3** [Å and °].

D-H···A	d(D-H)	d(H···A)	d(D···A)	<(DHA)
O(1H)-H(1H)···O(3W)	1.00	1.88	2.858(13)	165.1

Symmetry transformations used to generate equivalent atoms:

I: -x, -y+2, z **II:** -x+1, -y+2, z **III:** -x+1/2, y+1/2, -z

IV: x, y+1, z **V:** x, y-1, z **VI:** -x+1/2, y-1/2, -z

*S5.4 Bond length tables for Compound **4***

Table 6: Selected bond lengths [Å] and angles [°] for Compound **4**.

Mn(1)-O(1)^{III}	2.1451(15)	O(4)^{IV}-Mn(1)-O(4)^{V}	180.0
Mn(1)-O(1)	2.1452(15)	O(1)^{III}-Mn(1)-O(3)^{III}	95.27(6)
Mn(1)-O(4)^{IV}	2.1616(15)	O(1)-Mn(1)-O(3)^{III}	84.73(6)
Mn(1)-O(3)	2.2201(15)	O(4)^{IV}-Mn(1)-O(3)^{III}	84.44(6)
O(4)-Mn(1)^{VI}	2.1615(15)	O(4)^{V}-Mn(1)-O(3)^{III}	95.56(6)
		O(1)^{III}-Mn(1)-O(3)	84.73(6)
O(1)^{III}-Mn(1)-O(1)	180.00(8)	O(1)-Mn(1)-O(3)	95.27(6)
O(1)^{III}-Mn(1)-O(4)^{IV}	89.94(6)	O(4)^{IV}-Mn(1)-O(3)	95.56(6)
O(1)-Mn(1)-O(4)^{IV}	90.06(6)	O(4)^{V}-Mn(1)-O(3)	84.44(6)
O(1)^{III}-Mn(1)-O(4)^{V}	90.06(6)	O(3)^{III}-Mn(1)-O(3)	180.0
O(1)-Mn(1)-O(4)^{V}	89.94(6)		

Symmetry transformations used to generate equivalent atoms:
I: -x+1, y+1/2, -z-1/2 **II:** -x+1, y-1/2, -z-1/2 **III:** -x, -y+1, -z
IV: -x+1, -y+1, -z **V:** x-1, y, z **VI:** x+1, y, z

Table 7: Hydrogen bonds for Compound **4** [Å and °].

D-H···A	d(D-H)	d(H···A)	d(D···A)	<(DHA)
O(2)-H(2)···O(3)^{III}	0.84	1.82	2.647(2)	167.5

Symmetry transformations used to generate equivalent atoms:
I: -x+1, y+1/2, -z-1/2 **II:** -x+1, y-1/2, -z-1/2 **III:** -x, -y+1, -z
IV: -x+1, -y+1, -z **V:** x-1, y, z **VI:** x+1, y, z

*S5.5 Bond length tables for Compound **5***

Table 8: Selected bond lengths [Å] and angles [°] for Compound **5**.

O(1)-Mn(2)^{II}	2.1883(12)	O(3)^{I}-Mn(1)-O(1)^{III}	127.32(5)
O(1)-Mn(1)^{III}	2.2925(12)	O(1E)-Mn(1)-O(1)^{III}	83.69(5)
O(1E)-Mn(1)	2.2132(14)	O(2)-Mn(1)-O(4)^{I}	154.76(5)
O(2)-Mn(1)	2.1292(13)	O(5)^{V}-Mn(1)-O(4)^{I}	93.45(5)
O(2)-Mn(1)^{III}	2.4850(13)	O(3)^{I}-Mn(1)-O(4)^{I}	57.08(5)
O(3)-Mn(1)^{I}	2.1885(13)	O(1E)-Mn(1)-O(4)^{I}	105.36(5)
O(4)-Mn(2)	2.1270(13)	O(1)^{III}-Mn(1)-O(4)^{I}	72.56(4)
O(4)-Mn(1)^{I}	2.3809(14)	O(2)-Mn(1)-O(2)^{III}	78.68(5)
O(5)-Mn(1)^{IV}	2.1422(13)	O(5)^{V}-Mn(1)-O(2)^{III}	82.88(5)
O(6)-Mn(2)	2.1435(13)	O(3)^{I}-Mn(1)-O(2)^{III}	173.98(5)
Mn(2)-O(4)^{VI}	2.1269(13)	O(1E)-Mn(1)-O(2)^{III}	79.52(5)
Mn(2)-O(1)^{VII}	2.1884(12)	O(1)^{III}-Mn(1)-O(2)^{III}	54.41(4)
Mn(2)-O(1)^{VIII}	2.1884(12)	O(4)^{I}-Mn(1)-O(2)^{III}	126.14(4)
		O(4)^{VI}-Mn(2)-O(4)	180.0
Mn(2)^{II}-O(1)-Mn(1)^{III}	96.97(5)	O(4)-Mn(2)-O(6)^{VI}	90.53(5)
Mn(1)-O(2)-Mn(1)^{III}	101.32(5)	O(4)^{VI}-Mn(2)-O(6)	90.53(5)
Mn(2)-O(4)-Mn(1)^{I}	96.06(5)	O(4)-Mn(2)-O(6)	89.47(5)
O(2)-Mn(1)-O(5)^{V}	84.57(5)	O(6)^{VI}-Mn(2)-O(6)	180.0
O(2)-Mn(1)-O(3)^{I}	98.69(5)	O(4)^{VI}-Mn(2)-O(1)^{VII}	100.27(5)
O(5)^{V}-Mn(1)-O(3)^{I}	102.35(5)	O(4)-Mn(2)-O(1)^{VII}	79.73(5)
O(2)-Mn(1)-O(1E)	81.78(5)	O(6)^{VI}-Mn(2)-O(1)^{VII}	92.34(5)
O(5)^{V}-Mn(1)-O(1E)	159.52(5)	O(6)-Mn(2)-O(1)^{VII}	87.66(5)
O(3)^{I}-Mn(1)-O(1E)	94.78(5)	O(4)-Mn(2)-O(1)^{VIII}	100.27(5)
O(2)-Mn(1)-O(1)^{III}	132.67(5)	O(6)-Mn(2)-O(1)^{VIII}	92.34(5)
O(5)^{V}-Mn(1)-O(1)^{III}	94.55(5)	O(1)^{VII}-Mn(2)-O(1)^{VIII}	180.0

Symmetry transformations used to generate equivalent atoms:

I: -x, -y+2, -z+1 **II:** x, y+1, z **III:** -x, -y+3, -z+1

IV: x+1, y-1, z **V:** x-1, y+1, z **VI:** -x+1, -y+1, -z+1

VII: x, y-1, z **VIII:** -x+1, -y+2, -z+1

Table 9: Hydrogen bonds for Compound **5** [Å and °].

D-H···A	d(D-H)	d(H···A)	d(D···A)	<(DHA)
O(1E)-H(1E)···O(5)^{VIII}	0.886(16)	1.931(16)	2.8038(19)	168(2)
O(7)-H(7)···O(8)^{IX}	0.84	1.80	2.637(2)	176.5

Symmetry transformations used to generate equivalent atoms:

I: -x, -y+2, -z+1 **II:** x, y+1, z **III:** -x, -y+3, -z+1

IV: x+1, y-1, z **V:** x-1, y+1, z **VI:** -x+1, -y+1, -z+1

VII: x, y-1, z **VIII:** -x+1, -y+2, -z+1 **IX:** -x+1, -y+1, -z
